# Uncovering the eruptive patterns of the 2019 double paroxysm eruption crisis of Stromboli volcano

**DOI:** 10.1038/s41467-021-24420-1

**Published:** 2021-07-09

**Authors:** Daniele Andronico, Elisabetta Del Bello, Claudia D’Oriano, Patrizia Landi, Federica Pardini, Piergiorgio Scarlato, Mattia de’ Michieli Vitturi, Jacopo Taddeucci, Antonino Cristaldi, Francesco Ciancitto, Francesco Pennacchia, Tullio Ricci, Federico Valentini

**Affiliations:** 1 Istituto Nazionale di Geofisica e Vulcanologia-Osservatorio Etneo, Sezione di Catania, Catania, Italy; 2 Istituto Nazionale di Geofisica e Vulcanologia - Sezione di Roma 1, Roma, Italy; 3 Istituto Nazionale di Geofisica e Vulcanologia - Sezione di Pisa, Pisa, Italy; 4 Department of Geology, University at Buffalo, Buffalo New York, USA; 5 Dipartimento di Scienze, Università di Roma Tre, Roma, Italy; 6 Piazza Santa Maria Ausiliatrice, 10, Roma, Italy

**Keywords:** Petrology, Volcanology

## Abstract

In 2019, Stromboli volcano experienced one of the most violent eruptive crises in the last hundred years. Two paroxysmal explosions interrupted the ‘normal’ mild explosive activity during the tourist season. Here we integrate visual and field observations, textural and chemical data of eruptive products, and numerical simulations to analyze the eruptive patterns leading to the paroxysmal explosions. Heralded by 24 days of intensified normal activity and 45 min of lava outpouring, on 3 July a paroxysm ejected ~6 × 10^7^ kg of bombs, lapilli and ash up to 6 km high, damaging the monitoring network and falling towards SW on the inhabited areas. Intensified activity continued until the less energetic, 28 August paroxysm, which dispersed tephra mainly towards NE. We argue that all paroxysms at Stromboli share a common pre-eruptive weeks-to months-long unrest phase, marking the perturbation of the magmatic system. Our analysis points to an urgent implementation of volcanic monitoring at Stromboli to detect such long-term precursors.

## Abstract (web summary)

Integrated field and laboratory investigations, and numerical simulations of the 2019 paroxysmal explosions at Stromboli volcano revealed that they were anticipated by a week-to-month-long destabilization in the normal volcanic activity, a pattern common to all paroxysms.

## Introduction

**Fig. 1:**
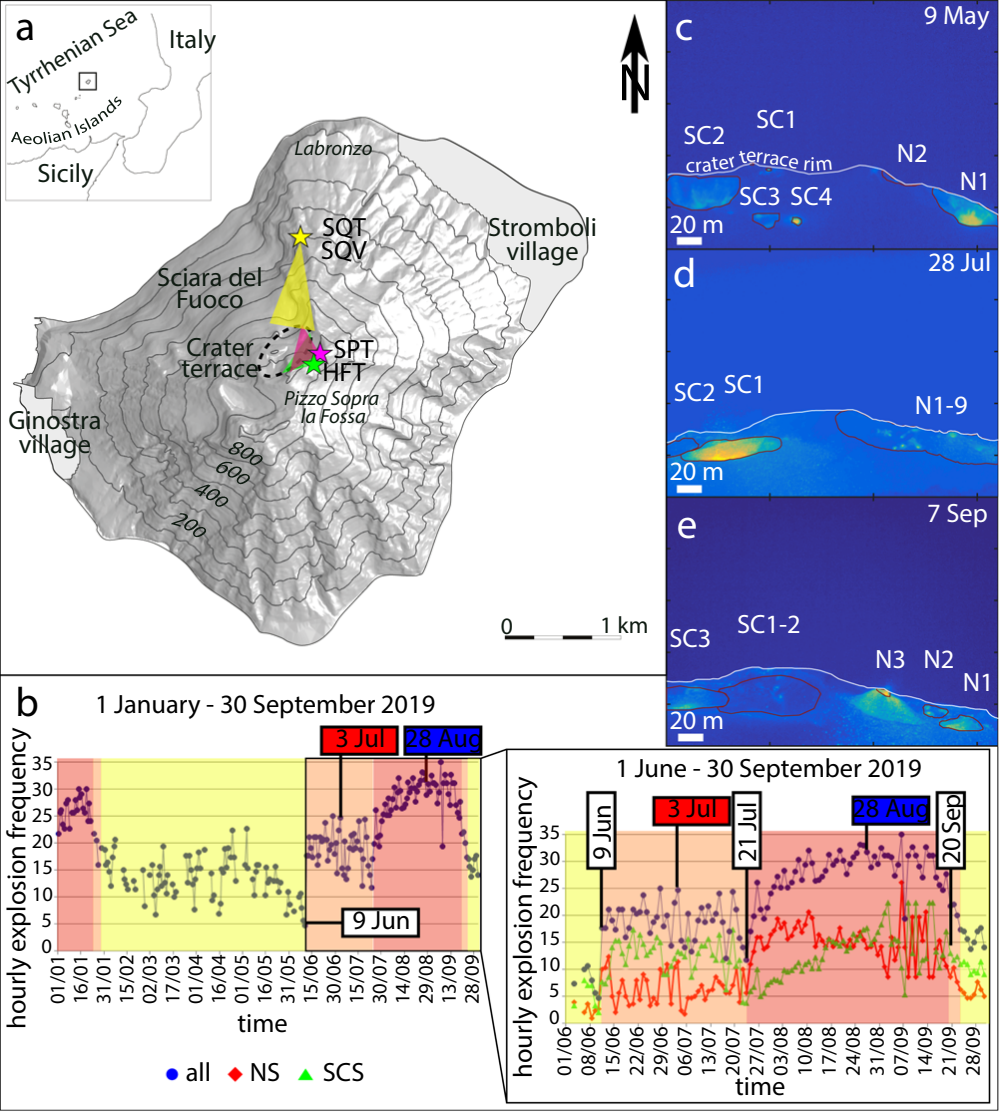
Map of Stromboli Island and location of the explosive activity. **a** Map of Stromboli Island with the location of Stromboli Pizzo Thermal surveillance camera (SPT, magenta star), portable high-frequency thermal camera (HFT, green star) at Pizzo Sopra la Fossa (918 m a.s.l.), and the Stromboli ‘Quattrocento’ (~400 m a.s.l.) Thermal and Visible cameras (SQT and SQV, yellow star) along the Sciara del Fuoco. The field of view of the crater terrace (dashed ellipse) of each camera is indicated by triangles. Gray areas: Stromboli and Ginostra villages. **b** Total hourly explosive frequency (HEF) per day in the period 1 January-30 September 2019, zoomed in the period 1 June-30 September for North Sector (NS) and South-Central Sector (SCS), respectively. Colored areas denote periods of medium (HEF ranging 5–15, yellow), high (HEF 15–25, orange), or very high (HEF > 25, red) activity levels. **c**, **d**, **e** Still frames from HFT video surveys from Pizzo on 9 May, 28 July and 7 September 2019, respectively, showing the morphology variations due to the two paroxysms and color-coded for the temperature scale (from blue to yellow tones marking increasing relative temperature). Numbers after letters indicate the different active vents in the N and SC sectors.

Active volcanoes are amongst the most captivating expressions of nature’s power. Numerous visitors and tourists approach active volcanic areas, attracted by the extraordinary beauty of their ongoing eruptive activity. However, the risk that an unexpected eruptive crisis can impact residents and visitors is always present, as tragically exemplified by the December 2019 eruption of White Island^1^ (New Zealand’s North Island). Such is the case of Stromboli Volcano (924 m a.s.l.; Fig. 1a), a small island of the Aeolian archipelago (Italy) and one of the most active and visited volcanoes in the world. Two villages, Stromboli and Ginostra, located on the NE and SW slopes, respectively, and only less than 2.5 km away from the active craters, count less than 500 residents in the winter. During the high season (July–August), the island can host up to 5000 tourists per day, either staying in holiday accommodations or arriving with daily boat tours. At the only pier of Stromboli, up to ten hydrofoils arrive per day and three ferry ships weekly. Dozens of boats sail along the seacoast at dusk to view the Strombolian explosions, and finally, mountain guides accompany an average of 200–300 hikers to the summit every day. While Stromboli’s inhabitants have learned to cohabit with the volcano and its sudden shifts to more violent behavior, the tourists remained shocked when two violent, larger than normal, paroxysmal explosions took place consecutively at the island in 2019, on 3 July and, after less than 2 months, on 28 August, with considerable hazard implications for the villages. After 3 July, Italian Civil Protection Department prohibited climbing the volcano, and until now, the summit remains off-limits with obvious effects on the tourist-based economy. Paroxysms at Stromboli are relatively rare events (34 in the last 140 years^2^). A recurrence of fewer than 2 months between consecutive paroxysms is even rarer and was reported to have occurred five times in the last century^2^. However, no monitoring system existed on the volcano until after the early Nineties, and the only other paroxysmal explosions studied with modern volcanological tools occurred on 5 April 2003 and 15 March 2007. In this regard, the 2019 crisis provided the first opportunity to investigate the close occurrence of two paroxysms, integrating data from a modern monitoring network, field-based observations, petrological measurements, and social media information, with numerical simulations.

At Stromboli, a persistent, normal eruptive activity has been ongoing since the 8th century AD^3^. The normal activity consists of mild to moderate Strombolian explosions, continuous degassing, and regular gas explosions or “puffing”^4^ occurring at a summit crater terrace. Explosion rate, style, and intensity are highly variable at the scale of a few hours or days, ranging between 0 to >25 explosions per hour (Supplementary Table 1). Volcanic jets^5^ can eject bombs to 10–150 m above the vents^6,7^ and have volumes of the erupted products ranging 1–10 m^3^ ^4^. In the last 140 years, when a consistent historical observation of the activity exists, the normal activity has been periodically interrupted by higher energy explosions, with an average frequency ranging from 0.7 to 2.1 events per year^2^, conventionally classified as major explosions and paroxysms^8,9^. Lava effusion phases, lasting days to months, have occurred with an average rate of 3.7 events per year from 1888 to 1986^9^.

Paroxysmal events pose an evident hazard for the inhabited areas, as they erupt more material (>10^4^ m^3^ in volume) to greater heights (>3 km above the vents), with volcanic ballistic projectiles ejected to distances of 1–3 km radially outward, and tephra covering the coastline and beyond^4^. The normal activity typically erupts crystal-rich (45–55 vol.%), black scoriaceous products, fed by a shallow, outgassed highly porphyritic (HP) basaltic magma residing at depths within about 2–4 km^10–13^. Conversely, paroxysms and most major events additionally release crystal-poor (<10 vol.%), brownish-yellow pumiceous-like products, related to a volatile-rich, low porphyritic (LP) basaltic magma rising from 7–10 km of depth^10,11,13^.

A current view is that the ascent and bursting of large gas pockets (or slugs) is driving explosions at all scales at Stromboli. The magnitude of explosions scales with the mass and separation depth of gas from the magma source involved, with no physically-based thresholds separating normal, major, and paroxysmal events^8,14–18^. The rapid, volatile exsolution-driven ascent, decompression, and fragmentation of LP magma, mingling with the HP magma during its rise, is also thought to be a trigger of the paroxysmal activity^10,13,19,20^.

In this work, in-depth scrutiny of the eruptive activity before and during the two paroxysms was carried out using data from: (i) surveillance video-recordings, (ii) high-frequency thermal camera video-recordings, (iii) images from social media, (iv) field studies of eruption deposits, (v) vesicularity analysis in lapilli-sized and ash-sized products, and (vi) chemical composition of erupted products. Data interpretation and numerical simulations provide answers on how the two paroxysmal explosions resulted from perturbations of the magmatic system feeding the so-called ‘normal’ explosive activity of Stromboli. The main scientific goal of this study is to use these integrated data to infer relatively long-term precursory signals of such hazardous events that apparently occurred unexpectedly.

## Results and discussion

### The 2019 eruptive activity as seen from videos

Before 3 July, the daily averaged hourly explosive frequency (HEF, Fig. 1b), ranged from very high in January (HEF up to 30, Supplementary Table 1), to low (HEF < 5) in early June. On 9 May, a survey using a portable high-frequency thermal camera (hereafter HFT) on the volcano summit area Pizzo Sopra La Fossa (hereafter Pizzo, 250–300 m above and SE of the active vents; Fig. 1a), indicated that the explosive frequency was medium (5–15 events per hour), and located at four active vents, two in the North Sector (NS) of the crater terrace, and two in the South-Central Sector (SCS), respectively, producing ash- and bomb-loaded jet-like explosions reaching 80–150 m elevations, i.e., in the medium intensity range (Fig. 1c, and Supplementary Movie 1). On 9 June, HEF increased abruptly to high (15–25 events per hour) and persisted at this level throughout June, producing up to 200 m high explosions (high intensity), lapilli fallout at Pizzo, and frequent emission of fluidal magma fragments from the NS. On 25 June, a major explosion occurred at vents SC1-SC2.

**Fig. 2:**
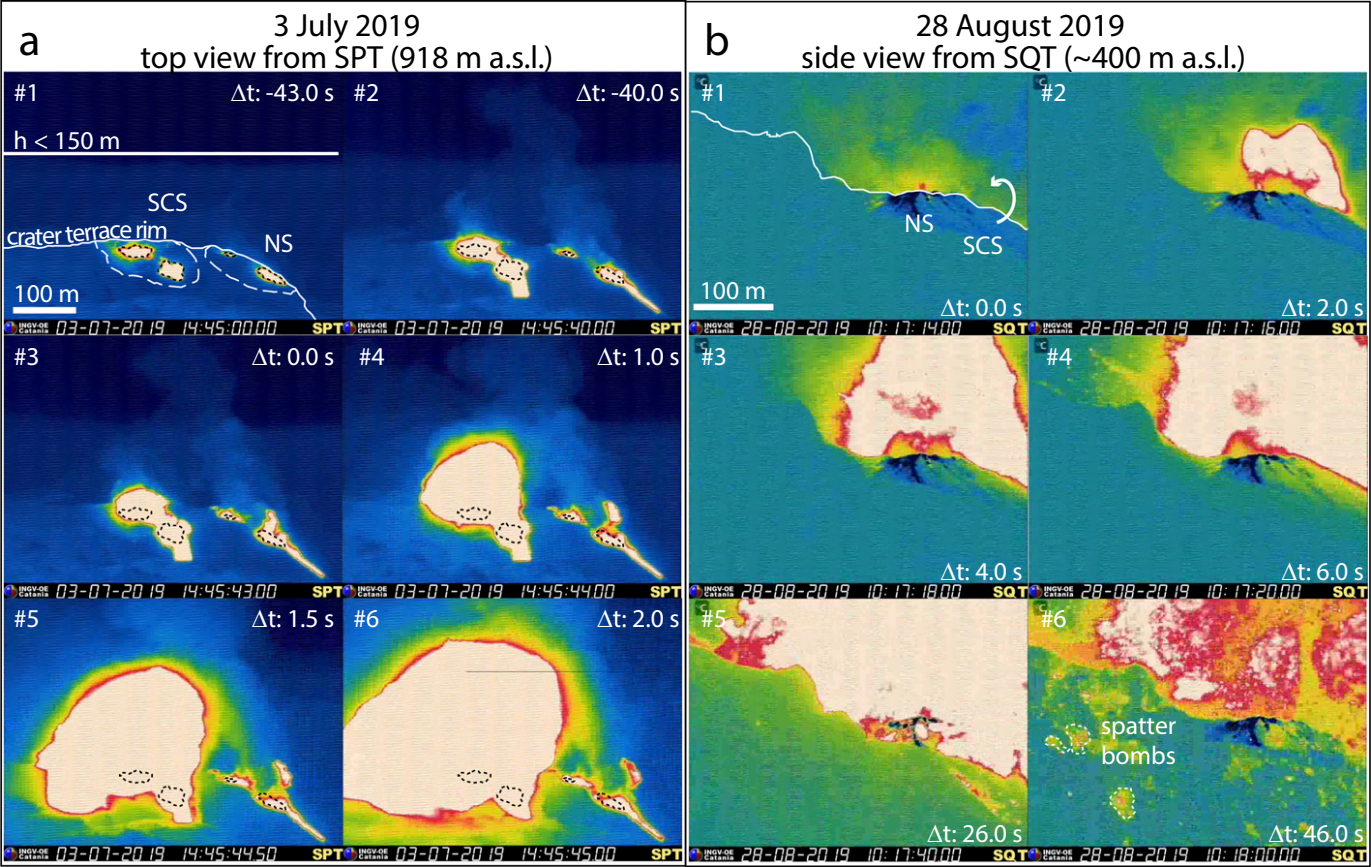
Thermal image sequences of the two paroxysms. Δ*t* indicates time difference from explosion onset. **a** 3 July paroxysm (from SPT camera): #1–#2 fast outflowing of lava occurs simultaneously at the North Sector (NS) and South-Central Sector (SCS), respectively, ~43 s before the paroxysm; #3–#6) explosion onset (at 14:45:43) and radial expansion of the incandescent jet in the following 2 s (14:45:45). For reference, the maximum elevation for ‘normal’ explosions is reported in #1. **b** 28 August paroxysm (from SQT camera): #1–#5 the progression of the initial blast between 1 s and 26 s after the explosion onset; #6) the flank of Stromboli covered by ‘spatter’ bombs (i.e., fluidal fragments of magma, dashed white lines) 46 s after the paroxysm.

On 3 July 2019, at 12:45 (all times are in UTC), i.e., ~2 h before the paroxysm, HEF exceeded 30 events per hour (very high), and after 13:20, ~85 min before the paroxysm, it was accompanied at vent N2 by a remarkable change in eruptive style from jet-like to violent spattering. At ~14:00:38, i.e., ~45 min before the paroxysm, another uncommon event occurred: a small lava flow pierced the terrace wall close to vent SC1 and descended inside the Sciara del Fuoco (hereafter Sciara; Fig. 1 and Supplementary Fig. 1). At 14:43:16, i.e., ~2 min before the paroxysm, the small flow suddenly slowed down, while the outpouring of lava started simultaneously at almost all vents (Fig. 2a and Supplementary Movie 2). The surface of the lava above the vents progressively inflated until, at 14:45:43, a spherical magma blast expanded radially from the SCS. Frame-by-frame tracking of the position of the incandescent front from SPT video revealed that velocity of the lava inflation in the 43 sec before the explosion (i.e., from 14:45:00 to 14:45:43) ranged ~2–30 m s^−1^, while the spherical front accelerated vertically to nearly 200–250 m s^−1^ in the first 2 s after the explosion (from 14:45:43 to 14:45:45, Table 1). After this time, the explosion saturated the ~500 m-wide and ~400 m-high field of view of the SPT camera, which transmitted the last frames discontinuously before destruction (Fig. 2a and Supplementary Fig. 1). The SQT camera showed that this first blast was followed 2 s later by a second blast from the NS (Supplementary Fig. 1). From this point of view, the mean vertical velocity of the radially expanding blast was ~91–103 m s^−1^ in the first 2 s of the explosion (Table 1). The SQV camera showed that after 3 s from the initial blasts, a shower of bombs landed on the Sciara for 14 s (from 14:45:46 to 14:46:00), and then a dense ash cloud expanded radially, obscuring the camera at 14:46:10, i.e., 27 s after the explosion onset.

**Table 1:** Main physical parameters of 2019 paroxysmal eruptions.

Paroxysm date			3 July 2019	28 August 2019	5 April 2003	15 March 2007
Total erupted mass (fallout deposits)	kg	Field data	5.9 × 10^7^ (this study)	/	1.1–1.4 × 10^8^ (Rosi et al.)^54^ (Pistolesi et al.)^33^	2.2–2.7 × 10^7^ (Pistolesi et. al.)^34^
			2.0–3.0 × 10^8^ (Métrich et al.) ^31^	/	/	1.9 × 10^7^ (Andronico et al.)^55^
		Simulated data	*7 × 10^7^ **4.2 × 10^7^ (this study)	/	/	/
MER	kg s^−1^	Field data	2.9 × 10^6^ (this study)	/	1.0–1.2 × 10^7^ (Rosi et al.)^54^ (Pistolesi et al.)^33^	1.9–2.3 × 10^6^ (Pistolesi et al.)^34^
			1.1 × 10^6^ (Giordano and De Astis)^32^	/	/	5.4 × 10^5^ ^55^ (this study)
		Simulated data	2.4 × 10^5^ (this study)	/	/	/
Plume initial velocity (first 2 s)	m s^−1^		SPT: 200–250 m/s SQT: 91–103 m/s (this study)	SQT: 55–59 m/s (this study)		
Plume velocity at max. elevation	m s^−1^		21–22 m/s (this study)			
Solid fraction density (DRE)	kg m^−3^		2772 ± 12 (this study)	2652 ± 55 (this study)	2860 (Pistolesi et al.)^33^	2850 ± 40 (Pistolesi et al.)^34^
Bulk density of lapilli	kg m^−3^	Mode	558–847 (this study)	352–602 (this study)	/	/
		Min–max	290–1512 (this study)	221–1615 (this study)	580–1840 (Pistolesi et al.)^33^	260–1080 (Pistolesi et al.)^34^
		Mean	760 ± 48 (this study)	500 ± 118 (this study)	1080 ± 250 (Pistolesi et al.)^33^	600 ± 170 (Pistolesi et al.)^34^
Lapilli vesicularity range	Vol. %	Mode	69–80 (this study)	77–87 (this study)	/	/
		Min–max	45–90 (this study)	39–92 (this study)	35–79 (Pistolesi et al.)^33^	65–95 (Pistolesi et al.)^34^
		Mean	73 ± 2 (this study)	81 ± 4 (this study)	61 (Pistolesi et al.)^33^	85 (Pistolesi et al.)^34^

We estimated the total erupted mass of the deposits and Mass Eruption Rate (MER) only for the 3 July 2019 paroxysm, either from field data or from numerical simulations. In this second case, we computed either the total erupted mass (*), or the mass estimated to be erupted on the island only (**). Available published data are reported for comparison: (i) erupted mass, converted from volume (3.53 × 10^5^ m^3^) using our estimated bulk density of the lapilli, and MER for the 3 July paroxysm;^31,32^ (ii) erupted mass, MER, lapilli density, and vesicularity, and DRE data for the 5 April 2003^33,54^ and 15 March 2007^34,55^ paroxysms.

Social media videos^21^ and images analysis revealed that the 3 July eruption column rose to ~6 km above the volcano summit, forming a volcanic plume that dispersed tephra on the ground in downwind directions. The plume rise velocity rapidly decreased from 120 to 44 m s^−1^ in the first kilometer, and then slowed from 35 to 22 m s^−1^ above that height.

After 3 July, the HEF remained at high levels until 22 July (12–25 events per hour), then progressively increased to very high levels (HEF > 30) until the second paroxysm (Fig. 1b). The first paroxysm induced important changes in the crater terrace morphology, as revealed by a 28 July rapid-response HFT camera survey (Fig. 1d and Supplementary Movie 3), showing nine simultaneously active vents in the NS and two in the SCS. The explosive intensity was high to very high (from 150 to >200 m elevation) and the eruptive style characterized by loud and very high-energy ash-rich and bomb-rich explosions that caused abundant ash fallout on Stromboli and Ginostra villages. Intermittent lava flows occurred from an isolated vent in the southern crater terrace but were confined to the upper Sciara (SC1-SC2 in Fig. 1d).

On 28 August at 10:17:14, a new paroxysm occurred (Fig. 2b, Supplementary Fig. 2, and Supplementary Movie 4). From the SQT camera, the blast initially rose with an estimated vertical velocity of ~55–59 m s^−1^ (Table 1), and after only 26 s from the explosion onset, the area above the Sciara was entirely saturated by the incandescent blast (Fig. 2b). Meter-sized fluidal fragments of magma were seen spreading widely in all directions. The SQV camera showed the formation of a jet and plume spreading mainly in a vertical direction and reaching the upper limit of the field of view (~300 m above the vent) in ~6 s. Bombs were ejected from the plume in all directions, forming ash fingers in their trails, and falling on the Sciara down to ~400 m a.s.l. elevation (Supplementary Movie 4). Following the second paroxysm, the explosive activity levels remained very high (25–35 events/hour) until waning from 20 September on (Fig. 1b). On 7 September, another rapid-response HFT survey showed high intensity (120–160 m elevation) and high frequency (20 events per hour) bomb-rich and ash-rich explosions occurring at six vents (three at the SCS, and three at the NS; Fig. 1e and Supplementary Movie 5). By 26 November, frequent ash emission had already covered the coarse-grained tephra deposit from the second paroxysm at Pizzo (Supplementary Fig. 3).

A quick analysis of social media provided an eruption column for the 28 August paroxysm rising up to ~6 km, consistent with that of the 3 July.

### Narrative of the hazardous phenomena accompanying and following the two paroxysms

A wide range of hazardous phenomena occurred during both paroxysms. During the two initial blasts, meter-sized bombs reached the NE volcano flanks down to an elevation of 600 m a.s.l (Supplementary Fig. 4), destroying the summit portion of the hiking trail. Shock waves induced by both paroxysms were strong enough to shatter glass and detach window frames in the villages (Supplementary Fig. 5). The damages to the monitoring network were numerous: the SPT camera, one FLux Automatic Measurement for SO_2_ monitoring (FLAME) station, two seismic stations, and one GPS station were destroyed or made unusable.

On 3 July, centimeter-sized, incandescent lapilli fell on Ginostra village for ∼30 min, with a suspended, dense ash cloud remaining for ~1 h between the summit and the lower flanks. The explosion suddenly induced two pyroclastic density currents (PDCs), formed by particle-laden jets collapse. The PDCs flowed down the NW flank of the volcano, reached the surface about 50 and 60 s after the explosion onset, and continued their run above the shoreline for almost 1 km. The entry of the PDCs into the sea caused a tsunami with a maximum wave height of about 40 cm at the coast and that was recorded instrumentally up to hundreds of km away^22^. The fallout of hot bombs occurred immediately after the explosion onset, lasted a few tens of seconds, and triggered bush fires that extended down to 500 m a.s.l. on the N side, while on the SW slope fires reached the uppermost houses of Ginostra (at 50 m a.s.l.). Those bush fires possibly caused the only casualty from the eruption: a hiker who was later found dead at ~200 m elevation above Ginostra. Tragic as this incident was, it could have proved a much greater tragedy, as, at the time of the paroxysm, hundreds of tourists had just started their ~3 h-long hikes to reach Pizzo.

On 28 August, tephra fallout dispersed to the NE side of the island affecting Stromboli village (Fig. 1a). Three minutes after the initial blast, a PDC descended the Sciara and into the sea, possibly due to column collapse. The resulting tsunami waves were ~60 cm high along the coast^23^, consistent with a fisherman reporting a wet strip on the rocks >0.5 m in the Ginostra harbor. Again, bomb-induced wildfires started from above the Stromboli village up to ~300 m elevation.

### Tephra dispersal and physical features of the deposits

**Fig. 3:**
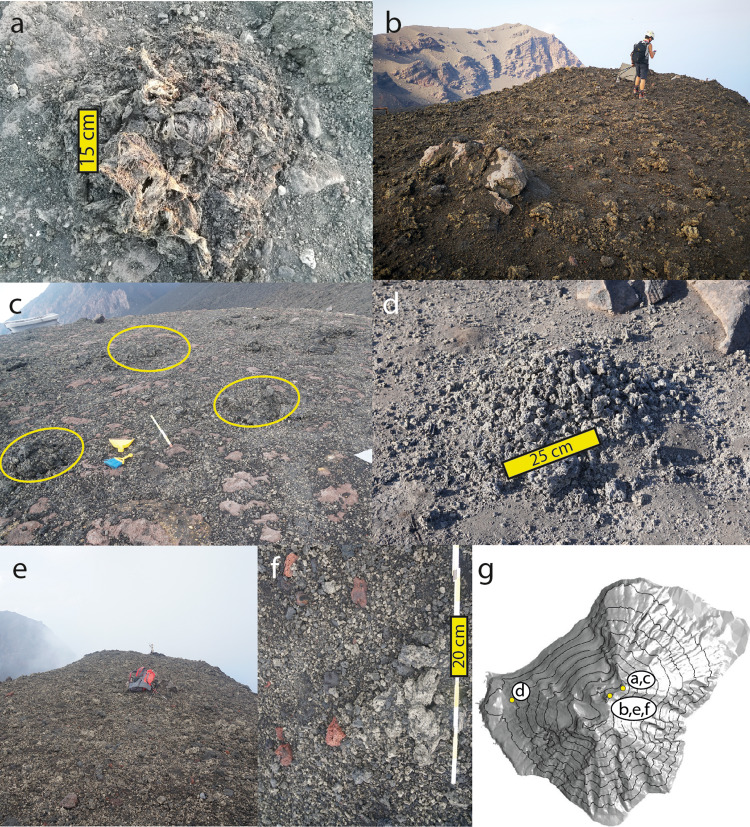
Impact of ballistics and tephra fallout after the paroxysms. **a**–**d** 3 July: (**a**) LP-dominated 0.6 m-sized spatter bomb resting above an HP bomb close to the summit trail, 450 m away from the craters; (**b**) continuous spatter and tephra coverage at Pizzo after 3 July; (**c**) discontinuous spatters coverage close to (**a**); (**d**) decimeter-sized bomb broken upon impact close to Ginostra village. **e**–**f** 28 August: (**e**) Continuous tephra fallout coverage at Pizzo after paroxysm; (**f**) detail of the deposit at Pizzo, showing unsorted fallout deposit, mainly made of pumices and non-juvenile fragments (reddish blocks and subvolcanic material); (**g**) map indicating the location of field observations. Photos by F. Ciancitto (**a**) and D. Andronico (**b**–**f**).

On the Pizzo area, the 3 July paroxysm deposited a 0.3–1 m (0.4 m average) thick continuous deposit, composed of a partially welded base of decimeter-sized to meter-sized ‘spatter’ bombs (i.e., flattened fragments still fluidal upon landing) of HP composition, upon which a discontinuous layer of 0.5–3 m-sized spatter bombs of LP composition was laid (Fig. 3a, b and Supplementary Fig. 4). The accumulation of abundant, decimeter-sized HP spatters on the steep NE slope of Pizzo generated a gravity-induced flow (similar to those described in a previous study^24^), a few meters wide and up to 0.5 m-thick, flowing down the E flank to 600 m a.s.l. (Supplementary Fig. 6). Descending from Pizzo towards NE, the spatter bombs cover became discontinuous below 850 m a.s.l. down to an areal dispersion of ca. 0.25 spatter m^−2^ at a 450 m distance from the vents, and also smaller in size (0.4–1.6 m, Fig. 3c and Supplementary Table 2); scattered spatter bombs were found down to 600 m a.s.l. Descending from Pizzo towards NW, fusiform (i.e., solidified upon landing) HP bombs became progressively dominant upon spatter ones and were found down to 300 m a.s.l. (Supplementary Fig. 4).

**Fig. 4:**
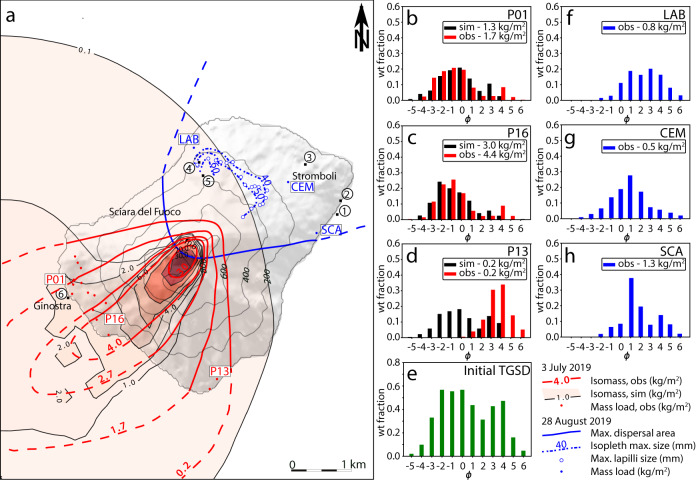
Dispersal of tephra fallouts from both paroxysms. **a** The mass load of the fallout deposit of 3 July measured at 16 locations (red points), and observed (red lines) and simulated (black lines contouring pink-shaded areas, modeled by PLUME-MoM-TSM/HYSPLIT) isomasses. The deposit is simulated at 18:00 UTC (more than three hours after the explosion) and reveals a maximum ground load of about 30 kg m^−2^. Circled numbers indicate 6 high-risk sites considered in the discussion. Estimated dispersal area (blue lines), and isopleths of the maximum lapilli size (dotted blue lines, see “Methods” section) of the 28 August fallout deposit. Isopleths were interpolated based on data collected from 17 locations (blue circles; Supplementary Table 3). **b**–**e** Comparison of simulated (sim) and observed (obs) grain-size distributions at three sampling points (P01, P13, and P16), and Initial Total Grain-Size Distribution (TGSD) at the volcanic vent resulting from the inversion of the data for the 3 July paroxysm. **f**–**h** Observed grain-size distribution of three samples of the 28 August tephra fallout (LAB, CEM, SCA); on the basis of observations of the eruption by a witness, the sample collected at Labronzo is affected by the contribution of fine ash due to the elutriated cloud produced by the PDC.

Tephra fallout deposits from the eruption plume were poorly sorted, ranging from ash-sized to bomb-sized pyroclasts, and were dispersed towards SW. A continuous, coarse-grained (10–50 cm) pumice bombs deposit, up to 0.7 m thick in the proximal sector, covered the upper W flank down to 500 m a.s.l. (Supplementary Fig. 6). N of Ginostra, at 100 m a.s.l., large (20–40 cm) pumiceous bombs, often broken upon impact, were found with an areal dispersion of 1–4 × 10^−2^ bomb m^−2^ (Fig. 3d); a few of them impacted the upper houses. In the village, the largest clasts ranged 3.7–12 cm (7 cm average), decreasing in size from N to S. The total erupted mass of tephra fallout was estimated from the isomass map to be 5.9 × 10^7^ kg (Fig. 4a, Table 1).

The 28 August deposits were only partially mappable, due to rapid road/roof cleaning by residents and fast removal by rain. However, it was possible to estimate that at Pizzo the deposit was nearly continuous and up to ca. 0.2 m thick, and composed of 90–95% fresh juvenile LP-HP magma pyroclasts and of 5–10% recycled volcanic and sub-volcanic fragments, i.e., non-juvenile blocks (Fig. 3e, f). The juvenile pyroclasts were entirely composed of centimeter-sized to decimeter-sized (max 0.4 m) sub-rounded, vesicular bombs, either white-colored (80%) or dark-colored (20%) externally. The tephra deposit was recognizable down to 300–500 m a.s.l. to the NE. Sparse clasts ranging 3–6 cm in size were found down to 200 m a.s.l., and up to 10 cm in the NNE slopes. Sporadic HP spatter bombs up to 1 m in size were found at 600 m a.s.l. towards NNE, and both HP bombs and non-juvenile blocks (0.1–0.5 m) reached 400 m a.s.l. NE (Supplementary Fig. 4). The distribution of the largest lapilli (See Methods) revealed a dispersal axis of the fallout towards NNE, over an area in between Labronzo (N) and the south Stromboli village (E) (Fig. 4a). The distribution of the cumulative weight of the 15 heaviest clasts also indicates a NNE dispersal trend (Supplementary Table 3).

The tephra fallout of 3 July produced large proportions of fine ash as demonstrated by the bimodality of the grain-size distributions (See Methods), with the coarser mode at between −1 (2–4 mm) and 0 Φ (1–2 mm) and the finer at 4 Φ (0.063–0.125 mm) (Fig. 4b–d). Clasts are made up of 100% juvenile material in the −5 (32–64 mm) to −2 Φ size range; non-juvenile clasts (altered red blocks and gray-colored scoriae) and loose juvenile crystals (clinopyroxene, olivine, and plagioclase) occur starting from the −2 Φ (4–8 mm) and −1 Φ (2–4 mm) interval, respectively, and progressively increase their abundance from 1 to 19% and from 11 to 31%, respectively, as the particle size decreases down to 1 Φ (0.5–1 mm). The 28 August samples are finer-grained than the 3 July ones with the mode around 1 Φ (0.5–1 mm, Fig. 4f–h). In the deposits from both paroxysms, the juvenile material displays mingled portions of LP light-colored pumices and HP darker scoriae. The LP portions are often found on the outside of the clast, where the quenched glassy surface can showbread crust-like fractures. At the macroscopic scale, the transition from HP to LP portions can be highly irregular, with mingling occurring in variable proportions (Supplementary Fig. 4f–h). Unmingled fragments of LP, distinguished from the HP ones for their lighter color and, in BSE imagines, for a darker gray tone, become more frequent with decreasing particle size, starting from the −2 Φ size interval.

### Petrochemical features of the eruption products

**Fig. 5:**
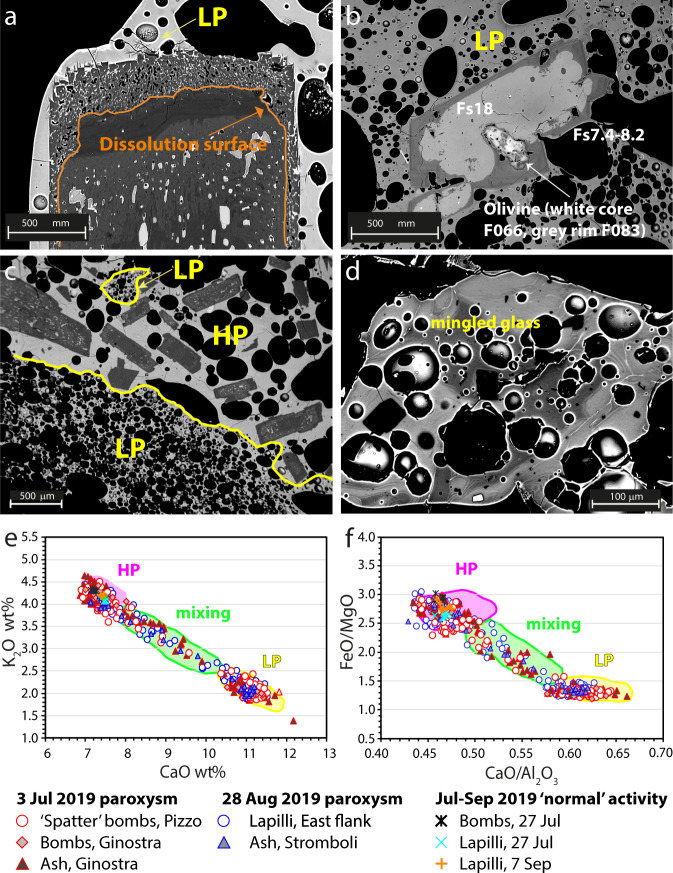
Petrographic and compositional features of minerals and glassy matrix in juvenile products of both paroxysms. **a**–**d** Backscattered images of minerals and groundmasses showing disequilibrium events evidenced by dissolution surfaces and crystal rims with variable textures and compositions: (**a**) corroded crystal of zoned plagioclase with a spongy textured corona; (**b**) clinopyroxene with Fe-rich corroded core (Fs18) and Fe-poor euhedral rim (Fs7.4-8.2), including a partially resorbed olivine (Fo66) with Mg-rich rim; (**c**) sharp contact between LP (dark-gray) and HP (light-gray) glasses in 3 July products; (**d**) highly mingled glass in 28 August products. **e**, **f** Chemical composition of glassy matrices plotted in CaO vs. K_2_O and CaO/Al_2_O_3_ vs. FeO/MgO diagrams. The compositional field of HP, LP, and mixed HP-LP products that erupted in the past two decades are reported for comparison (published^10,13,19,69^ and unpublished data).

At the microscopic scale, extensive mingling between LP and HP magmas in both paroxysms is highlighted by a marked chemical inhomogeneity in the glassy matrix and mineral chemistry, and complex textural and chemical zoning of the crystals. Among them, partially dissolved plagioclases, inherited from the HP magma, are quite common in LP pumices (Fig. 5). They are encircled by large skeletal-like anorthitic rims, resulting from a high growth rate due to rapid degassing^12,25^. In addition, many olivine and pyroxene crystals in LP clasts have partially dissolved Fe-rich cores (Fig. 5a, b), coming from an old crystal mush intersected by the LP magma during its ascent^26^. In the 3 July products, LP and HP portions have sharp contacts in the mingled pumices/scoriae, and glasses with intermediate compositions between HP and LP magmas are limited to 100–200 µm thick zones at the boundary between the two end-members (Fig. 5c). Conversely, in the 28 August products, the mingling appears more pervasive, as suggested by up to 400–500 µm wide inhomogeneous areas with abundant intermediate compositions (Fig. 5d). The compositions of the LP and HP matrix glasses (shoshonitic-basalts with K_2_O 1.85–2.35 wt.% and shoshonite with K_2_O 3.8–4.5 wt.%, respectively) fall in the compositional range typifying the products of the past two decades (Fig. 5e, f). Lapilli and ash fragments related to the inter-paroxysmal (27–28 July) and post-paroxysmal (7 September) activity fall in the field of HP products and are characterized by a homogeneous shoshonitic glass composition (K_2_O 4.1–4.2 wt.%).

### Petrophysical features of tephra particles

**Fig. 6:**
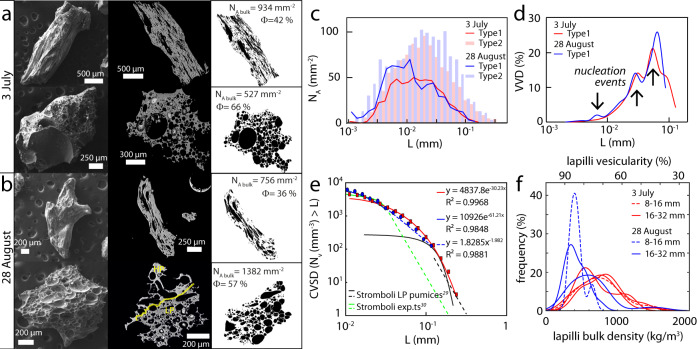
Morphological and physical features of ash and lapilli. **a**–**b** SEM images of selected LP clasts from the two paroxysms, showing external morphology and internal texture (left and center columns), and binary images (right column; vesicles in black with Φ = vesicle area fraction %, and *N*_A bulk_ = total number of vesicles per mm^−2^ within each class). **c** 2D distribution of the major axis of the best-fit ellipse (L) for Type1 (lines) and Type2 (bars) vesicles in number density (*N*_A_ mm^−2^). **d** 3D volume distribution of Type1 vesicles (VVD). Arrows point to nucleation events. **e** Log-plot of the cumulative vesicle size distribution (CVSD), i.e., the number density of 3D (N_v_, Type1) vesicles larger than L (major axis of the vesicle best-fit ellipse) against L. Equations of power-law (dashed) and exponential (solid) curves fitting the data are shown together with the R^2^. For comparison, published data on 0.01–1 mm vesicles data Stromboli 2003 and 2007 paroxysms^29^ (black lines) and experiments^30^ (green lines) are shown. **f** Density distribution of lapilli in the size range 8–16 and 16–32 mm and corresponding estimated vesicularity distributions for 3 July (red lines) and 28 August (blue lines, See “Methods” section).

The textural investigation was carried out on the ash fraction (particles < 2 mm) from both paroxysms. Ash analysis allows quantifying the rising processes in the conduit that are recorded in the groundmass and that are directly linked to the rising of the deep LP magma (See Methods). LP clasts are mostly ‘fluidal’^27^, i.e., elongated and with shiny and smooth external surfaces, and less commonly ‘spongy’^27^, i.e., vesicular fragments with nearly spherical bubbles of similar size intersecting the external surfaces of the clast (Fig. 6a, b). In thin sections, ash particles display a microlite-free groundmass with a large range of vesicularity (from 36 to 66 vol.%, Fig. 6a, b). HP particles show fluidal external surfaces, and often embed plagioclase phenocrysts (up to 2 mm-long) in a highly vesicular (up to 1 mm-sized vesicle) groundmass (Supplementary Fig. 7).

Since the paroxysms are related to the LP magma ascent, an in-depth vesicularity investigation focused on this component. LP ash fragments display both round (aspect ratio, AR ≥ 0.8) and elongated (AR < 0.8), irregularly shaped vesicles, hereafter referred to as Type1 and Type2 vesicles, respectively (Supplementary Results and Supplementary Fig. 8). 2D vesicularity data show that in both paroxysms, Type1 vesicles are less abundant (11–13 vol.%, Table 2), and smaller (0.2–152 µm, mode at 12 µm, Fig. 6c) than Type2 ones (37–39 vol.%; 0.5–667 µm, mode at 20 µm). The 3D vesicularity was computed on Type1 vesicles only to avoid stereological problems during the conversion of the shape from 2D to 3D^28^ (see “Methods” section). The Vesicle Volume Distribution (VVD) shows that LP products of the 28 August paroxysm differ from the 3 July one for a larger proportion of smaller vesicles and both show the occurrence of multiple nucleation events (Fig. 6d, Table 2). The cumulative volumetric vesicle size distributions (CVSD, Fig. 6e) of Type1 vesicles follow two different trends, represented by an exponential-like curve for the 3 July event and a mixed power-law and exponential distribution for the 28 August one. Both paroxysms have vesicle number densities (*N*_v bulk_, Table 2) at least an order of magnitude larger than that of the 2003 and 2007 paroxysms^29^, but comparable to that of experimental simulations^30^.

**Table 2:** Vesicularity data in LP magma for the 2019 paroxysmal eruptions.

Paroxysm date		3 July 2019		28 August 2019		5 April 2003^29^	15 March 2007^29^
Vesicle type		Type1 (w/L > 0.8)	Type2 (w/L < 0.8)	Type1 (w/L > 0.8)	Type2 (w/L < 0.8)	/	/
Ref. area _melt_	mm^2^	7.17		2.09		/	/
*n*		2006	3741	715	1432	/	/
L (Peak size)	µm	8	20	4	16	/	/
L (Size range)	µm	0.2–152	0.5–667	0.5–122	1–365	/	/
*N*_A bulk_	mm^−2^	280 (50–500)	521 (135–1160)	342 (88–773)	685 (555–1255)	/	/
*N*_V bulk_	mm^−3^	4069		5540		270	376
Φ	area %	13 (0–27)	37 (24–50)	11 (0–30)	39 (35–43)	58	57

Ref. Area _melt_ stands for the sum of the area of studied LP clasts (corrected for vesicles) used for vesicles quantification; n is the number of vesicles counted in the analyzed area; Peak size is the main peak diameter in vesicle size distribution histogram; Size range is a whole range of vesicle diameters; *N*_A bulk_ is the number density of vesicles per unit of area (n/Ref. Area) (minimum-maximum values in brackets); *N*_V bulk_ is the number density of vesicles per unit of volume computed using CSD correction software^70^; Φ is the vesicles area fraction (minimum-maximum values in brackets). Available *N*_V_ and Φ data from literature^29^ on the 2003 and 2007 paroxysms are also reported for comparison.

Density measurements were carried out on −3 and −4 Φ lapilli-size particles (8–16 mm and 16–32 mm, see “Methods” section), showing mostly unimodal distributions peaked at modal values of 558–847 kg m^−3^ and 352–602 kg m^−3^ for the 3 July and 28 August products, respectively, with a tail of denser clasts (Fig. 6f). The solid fraction density (DRE) of crushed lapilli is higher for 3 July (2772 ± 12 kg m^−3^) than for 28 August (2652 ± 55 kg m^−3^) products. The corresponding calculated lapilli vesicularity distributions are peaked at modal values of 69–80% and 77–87%, respectively.

### Numerical retrieval of the eruptive source parameters

Numerical simulations were carried out to model the 3 July paroxysm only, for which robust field data were available. We integrated several numerical codes (See Supplementary Methods) to find the best fitting eruptive source parameters (total grain-size distribution and mass eruption rate at the vent) that minimize the difference between the simulated and the observed tephra fallout deposit at 16 locations, with distance from the vent ranging between 550 and 2450 m (Supplementary Fig. 9). The best-fitting procedure of the tephra fallout deposit produced a simulated load at all locations differing by a factor 0.4–2 from the observed ones (Fig. 4 and Supplementary Fig. 10), providing a time-averaged mass eruption rate of 2.43 × 10^5^ kg s^−1^. This results in a total erupted mass of 7.0 × 10^7^ kg, and a mass of 4.2 × 10^7^ kg (Table 1) deposited on the computational domain of the simulations (an area slightly larger than the island, including small areas of sea belonging to the domain), matching well with the value obtained from field data. Additional results are a maximum column height above the vent of 4.3 km, a total grain-size distribution at the vent with the coarser particles having a mode of −1 Φ, while the finest particles show a mode of 4 Φ (Fig. 4e), and a shape factor of 0.24 for particles with size Φ < 3 and 1 for finer particles. Almost 100% of the finest particles (6 Φ) reach the top of the plume, while about 70% of the coarser particles (−5 Φ) are lost along the plume margins—from its bottom to its top—due to their greater settling velocities. The simulations were not able to reproduce the observed grain-size distribution at the most proximal point (Supplementary Fig. 10) and the two southernmost points (Fig. 4d), due to the lower sampling density in these areas. Overall, the simulated tephra fallout poorly reproduced the secondary fine-grained mode of the 3 July grain-size distributions (Fig. 4). However, additional simulations where fine particles were released from a source area located at the vent height were able to better reproduce the deposition of fine particles (Supplementary Fig. 11).

### Comparing eruption parameters of paroxysmal explosions at Stromboli

The 3 July 2019 fallout deposit displays intermediate total erupted mass with respect to the two previously studied paroxysms of 5 April 2003 and 15 March 2007 (Table 1). Our estimated mass (5 × 10^7^ kg) for the fallout deposits is lower with respect to other similarly estimated values^31^ (Table 1). Our GSDs are consistent with previous ones^32^, both showing secondary fine ash modes. Mass eruption rates ranging from 2.4 × 10^5^ (simulation) to 3.6 × 10^6^ kg s^−1^ (observation), are in agreement with previous values^32^ (1.1 × 10^6^ kg s^−1^), although this was retrieved from the eruption column height. A similar^32^ estimated average jet velocity is obtained from the SQT camera (91–103 m s^−1^), although our frame-by-frame analysis from the SPT camera provides a better time-resolved velocity of 200–250 m s^−1^ in the first 2 s after the explosion onset. Our estimated plume height (~6 km) is slightly lower with respect to the ~8.4 km value found previously^32^. Regarding the 28 August paroxysm, the mass of selected tephra samples and the size of the largest clasts at comparable distances from the crater areas suggest that the magnitude of the eruption was lower than the 3 July one, despite the similar estimated plume height of 6 km (consistent with the previous values^32^). This apparent contradiction can be explained by the surveillance camera observations suggesting that the magma fragmentation level, marking the explosion onset, of the 3 July paroxysm was just outside the vent (Supplementary Fig. 1), i.e., in the atmosphere, whereas it was deeper in the 28 August paroxysm (Supplementary Fig. 2), possibly in the shallow conduit. Therefore, the unconfined expansion in the 3 July case had a relevant horizontal component, and thus the energy associated with the overpressured gas contributed only partially to the vertical acceleration of the jet. Conversely, in the 28 August case, the expansion occurring during the decompression to atmospheric conditions was confined laterally, resulting in a larger vertical acceleration of the initial jet. This led to a plume height comparable to that of 3 July, despite the smaller magnitude.

The bulk density and vesicularity of the lapilli (Table 1) match well those of the 2003 and 2007 paroxysms. The 3 July solid fraction density value is slightly lower but in agreement (within variability ranges) with previously measured values on LP particles^33,34^ (Table 1), while that of the 28 August is much lower. This could imply that a larger batch of low-density LP magma was involved in the two 2019 paroxysms, near to the low-density values calculated for the crystal-poor LP magma^13^. Regarding ash particles vesicularity, the distribution of the CVSDs (Fig. 6e and Table 2) suggests that all four paroxysms (2003, 2007, and 2019) share a similar process of continuous/accelerating bubble nucleation^29,35^, indicating that disequilibrium degassing occurred because the magma was ascending rapidly in the conduit. The slightly different, exponential-like trend (Fig. 6e) of the 3 July CVSD, suggests that the timing of magma ascent, in this case, allowed minor bubble growth and coalescence. The 2019 CVSDs have intermediate *N*_V bulk_ values between those experimentally reproduced at 1.5 m s^−1^ (consistent with the 2003 and 2007 paroxysms^29^) and 3 m s^−1^ ^30,36^ (Fig. 6e). This suggests that the magma ascent rates of the 2019 paroxysms were higher than those of 2003 and 2007, the 28 August probably ascending faster than the 3 July one (Fig. 6e and Table 2), in agreement with our reported evidence from visual observations on the explosion onset and initial jet development. Neglecting magma acceleration during ascent from depth to surface, these ascent rates provide average magma rise timescales between 14 and 21 min if the source depth of 1.8–2.4 km, inferred from tilt inflation, is considered^37^, and between 86 and 43 min for LP magma to ascent from a magma source depth of 7–8 km^36^. Strikingly, this latter timescale is coincident with the ~45 min of precursory lava outpouring before the 3 July 2019 paroxysm.

Petrochemical compositions overlap those of previous paroxysms (Fig. 5). The more extensive mixing between HP and LP magmas in the 28 August products compared to those of 3 July is apparently in contrast with the inferred faster ascent of the 28 August magma. However, the shallow magma system was largely destabilized after 3 July, showing frequent and intense explosive activity (Fig. 1), lava effusion, and enhanced gas supply from depth as suggested by monitoring data^38^. This could have caused an overall temperature increase of the shallow, ∼3 km deep, magma system, thus lowering the viscosity contrast between HP and LP magmas and allowing a more efficient magma mixing during the 28 August eruption compared to the 3 July one.

### Hazard implications from tephra fallout

The results of our numerical simulations allow estimating the time necessary to take action and protect people from tephra fallout at Stromboli and Ginostra villages. Simulated cumulative loads at 6 strategic locations at elevations <400 m a.s.l., including harbors, the school, the heliport, and tourist viewing points (1–6 in Fig. 4), provided 0.01 to 2.6 kg m^−2^, mainly in the particle classes −3–3 Φ (Supplementary Fig. 12). For all locations, simulated tephra deposition started within 5 min after the beginning of the paroxysm and 90% of the total load was deposited within ~20 min (Table 3). An additional simulation with the same eruptive parameters, but setting a hypothetical main wind direction towards the village of Stromboli, computed a comparable onset time for deposition at Stromboli village, i.e., ~5 min, and a deposited load of about 1.1–6.2 kg m^−2^ (90% deposited in 10–25 min; Table 3 and Supplementary Figs. 13 and 14). Regarding hazard, a ground load of this magnitude is not able to severely affect infrastructures in the villages, but a 5-min time span to get to safety requires a very prompt response and the accessibility of a shelter nearby, especially for protecting people from coarse-grained tephra fallout.

**Table 3:** Deposition timings and cumulative loads determined from numerical simulation of 3 July event at six high-risk locations.

Site features				Coordinates		3 July tephra fallout			NE tephra fallout		
Site number	Selected sites	Area	Type of use	Latitude	Longitude	Deposition start mins after explosion	Deposition end * mins after explosion	Ground load kg m^−2^	Deposition start mins after explosion	Deposition end * mins after explosion	Ground load kg m^−2^
1	Scari harbor	Stromboli village	Crowded/ meeting place	38.797533°	15.239998°	5	20 (20)	0.014	5	105 (25)	1.336
2	Scari helipad	Stromboli village	Meeting place	38.799549°	15.240411°	5	20 (20)	0.012	5	105 (25)	1.238
3	Football field-S. Vincenzo schoolhouse	Stromboli village	Student crowded/ meeting place	38.804834°	15.233797°	5	30 (20)	0.022	5	105 (20)	1.069
4	Viewpoint/trail-190 m	Above Labronzo	Tourist	38.805139°	15.213889°	5	35 (20)	0.156	5	110 (20)	1.615
5	Viewpoint/trail-400 m	Above Labronzo	tourist	38.803201°	15.214623°	5	25 (20)	0.166	<5	130 (10)	6.217
6	Porto Pertuso harbor	Ginostra village	Crowded/ meeting place	38.785361°	15.189310°	5	180 (20)	2.611	5	95 (20)	0.053

Scari harbor and helipad in Stromboli, Football field/S. Vincenzo schoolhouse, two viewing points/trails above Labronzo and Porto Pertuso harbor site locations (1–6) are reported in Fig. 4. For speculative purposes and hazard estimation, a tephra fallout simulation was also run with the same 3 July parameters but with an opposite wind direction (NE). In brackets are the minutes after the explosion for the deposition of 90% of the total load.

A further potential hazard is represented by a large amount of fine ash (<1 mm)^39^ produced by the eruption, in the order of 15–99% in terms of groundmass along the SW coastline (Fig. 4d). This has severe effects on human health since large quantities of PM_10_ (particles with dimensions ≤10 µm) in the air can be dangerous if inhaled^40^. Although we have not quantified ash particles <10 µm, the 6 Φ fraction (<32 µm) constituted ~2–61 g (average = 23.3 g) of the groundmass of tephra deposit per square meter in the most densely inhabited area of Ginostra. This cannot be explained only with the primary fragmentation of the ascending magma, and a more in-depth study is required to ascertain its origin, which could be linked, e.g., to the abrasion and comminution of juvenile particles during transport within a PDC^41^. However, our numerical simulations, as well as the presence of non-juvenile fragments in the fine deposit fraction, suggest that the additional source of finer particles is possibly associated with the partial disruption of the crater terrace, as also invoked, e.g., during caldera collapses at other volcanoes^42^. Another potential cause for the secondary maximum could be particle aggregation, inducing premature settling of fine ash particles^43^.

### Paroxysmal explosions at Stromboli: are they unpredictable?

It is known that Stromboli’s normal eruptive pattern is episodically interrupted by paroxysmal eruptions whose frequency and magnitude vary widely. Other persistently active volcanoes worldwide such as, e.g., Volcán de Fuego^44^ and Pacaya^45^ (Guatemala), Kilauea^46^ (Hawaii), Batu Tara^47^ (Indonesia), show similar, sudden shifts to more violent eruptive behavior. At Stromboli and Fuego, the influx of a hotter, volatile-rich, and primitive magma type and it’s consequent mingling/mixing with its cooler, degassed, and more evolved end-member is invoked as the mechanism triggering more violent eruptive behaviors^10,48,49^. It is however still unclear at Stromboli whether these events happen without prior notice or if they are preceded by reliable precursors in the normal activity. The small record of the occurrence of such violent episodes since the existence of a suitable monitoring network on the island (established in 2003), makes it difficult to statistically interpret the observational data. Recently, the a posteriori analysis of geochemical and geophysical parameters from the monitoring network revealed the long-term occurrence of a CO_2_ anomaly starting from ~two weeks and ~two months before the 15 March 2007 and 3 July 2019 paroxysms, respectively^50,51^, and of a VLP signal size anomaly one month before the 3 July paroxysm^52^. Furthermore, the re-analysis of tilt time series documented a short-term volcano inflationary pattern, started 2.4–3.4 min^53^ before the 3 July and the 28 August paroxysms. A more recent study asserts that such deformations were detectable from ~10 min^37^ prior to the last four paroxysmal explosions. These short timescales, also linked to the rapid degassing detected from lithium diffusion in plagioclase^53^, were interpreted as the only precursory signals prior to the two paroxysms, thus posing questions regarding the effective capability to forecast such kind of paroxysms in due time.

Our evidence on the 2019 paroxysms and data from the 2003 and 2007 ones suggest that the pre-eruptive changes in the intensity, frequency, and style of normal explosions patterns observed before paroxysms must effectively be considered as long-term precursory signals. In fact, the 2019 paroxysms occurred within a ~3-month-long period of high to very high HEF and explosive intensity, initiated nearly a month (9 June) before the 3 July paroxysm, in line with the onset time of the detected VLP size anomaly^52^, and waned nearly a month after the second one (28 August). Small effusions, limited in volume and confined to the upper Sciara, preceded the first eruption by 45 min and continued in the inter-paroxysmal period, indicating the magmatic system was still perturbed after 3 July, and possibly suggesting that the two paroxysmal explosions represent two episodic releases of the same deep magma input. Similarly, the 5 April 2003 and 15 March 2007 paroxysms were preceded by months-long periods of anomalous eruptive activity: first, the volcano underwent ~2 month-long and ~3 week-long phases of increased explosive frequency, respectively; second, at the crater terrace the explosive activity stopped and was replaced by an extensive effusive phase, lasting ~3 months and ~2 weeks, respectively^4,33,34,54,55^, occurring from eruptive fissures located in the NE flank of the volcano^56,57^. Both paroxysms occurred during the ongoing effusive phase, which still continued afterward, while the normal activity at the summit craters only resumed weeks to months later^56–58^.

Based on such evidence, we argue that 2003, 2007, and 2019 paroxysms were heralded by weeks-long to months-long periods of volcanic unrest, although an unambiguous eruptive pattern preceding a paroxysmal explosion cannot be recognized yet. In our view, the deep destabilization process in the magmatic system triggering the paroxysms occurs in three main stages: (i) it starts weeks to months before the events, with the refilling of LP magma and/or its CO_2_-rich gas phase in the crustal reservoir;^31,50,51^ (ii) it continues through increased volatiles emissions^50,51^, and LP magma interacting and mixing^10,31^ with the residing HP magma; and (iii) it culminates, hours to minutes^37,53^ before the events, with the final acceleration of a larger-than-normal volume of porous and vesicular LP/HP magma, leading to the onset of the paroxysmal eruptions. The first two stages are documented by the long-term (months to weeks) anomalies in the normal eruptive activity, as well as in the geophysical and geochemical signals, while the final stage of rapid ascent and decompression is recorded by the short-term ground deformations and precursory lava outpouring, and by the lithium diffusion in plagioclases, CVSD trend and high *N*_v_ registered in erupted products.

We conclude that during an unrest phase, the ability to correctly recognize precursors heralding changes in the magma system in the short (hours-minutes) and long (weeks-months) term is crucial for estimating the potential occurrence of such events. We think that such changes could be efficiently detected in the normal explosive activity by accurately monitoring the eruptive parameters (i.e., frequency, intensity, and style), and by running daily geochemical and textural analyses of eruptive products. In this way, the arrival of a new magma/volatile input can be identified and help constrain the evolution of the future activity, especially when integrated with long-term geophysical observations.

### A new eruptive cycle

The historical record indicated that from 1959 to 2003 Stromboli underwent a ~forty-year-long phase without paroxysms. In the last 25 years, an intensification of violent (major and paroxysmal) explosions resumed, with an average of 2.1 episodes per year^2^. Over the past seventeen years, four paroxysms occurred, two of which in just a few months. Starting from 2017, Stromboli’s explosive activity showed intermittent phases of increases in HEF and explosive intensity on two occasions, causing the Civil Protection Department to raise the alert level to yellow, i.e., indicating a state of potential disequilibrium of the volcano^59^. A clear change in the steady-state of the Stromboli plumbing system feeding the normal activity has been inferred by studies on clinopyroxene/melt equilibrium of the recent 2003–2017 activity^60^, indicating that magmatic injections feeding the persistent activity are more intensively mixed and homogenized prior to paroxysmal eruptions than in the past. We conclude that these studies, together with the recent increase in the frequency of paroxysmal events, could warn that a new eruptive cycle has opened, possibly similar to that of the 1880–1959 period where paroxysmal events averaged 0.4 per year^2^.

### Future challenges

Reconstructing the timeline of magma processes prior to paroxysms at Stromboli represents a considerable challenge for volcanic risk assessment and hazard mitigation. Understanding the dynamics and evolution of paroxysmal eruptions in 2019 has benefited from an integrated, in-depth analysis of a large and diverse data set. Such analysis revealed that the 2019 paroxysms were anticipated by a week-long destabilization in the normal activity. A comparison of the 2019 and previously studied paroxysms indicated that a pre-paroxysm change in the ordinary state of the volcanic activity was always present but in different forms (e.g., effusive activity, increased HEF, or normal explosions intensity), despite all paroxysms sharing the same eruption trigger, and similar dynamics, magnitude, and intensity. Today’s scientific challenge is to find the nexus between the changes in normal activity and the destabilization of the deep magmatic system that initiates the pre-paroxysm perturbation and culminates with a paroxysmal explosion. We speculate that innovative, high frequency petrological and volcanological monitoring, integrated with geophysical monitoring, will be key in revealing these precursory links.

## Methods

### Volcanic surveillance of Stromboli

The Istituto Nazionale di Geofisica e Vulcanologia (INGV) is a national research institute carrying out research, monitoring, and surveillance in the Italian volcanic areas through “Departments” located all over the national territory. It also supports the Italian Department of Civil Protection. Since 2001, INGV has been monitoring the volcanic activity of Stromboli, covering different research fields such as volcanology, petrology, gas geochemistry, seismology, ground deformations, and publishes weekly reports at www.ct.ingv.it. The monitoring system has been strengthened after each of the recent 2002–2003 and 2007 eruptions, both characterized by effusive activity which replaced the normal Strombolian activity, and punctuated by paroxysmal events on 5 April 2003 and 15 March 2007, respectively^33,34,54,55^.

### Video-camera recordings analysis

Before the 3 July paroxysm, the INGV-OE video-surveillance system at Stromboli consisted of three cameras acquiring and transmitting footage in real-time to the Control Room located in Catania: a thermal FLIR A320 at Pizzo (SPT), with Field of View (FOV) 90° × 73°, at ~250 m away from the crater terrace and working at 2 fps, and two cameras at 400 m a.s.l. (both at ~920 m away and 0.5 fps), i.e., a thermal FLIR A320 (SQT) with FOV 25° × 18°, and a visible Sony FCB-EX480CP, with 18 × 48° (wide end FOV) (Fig. 1). Using this footage, we observed and quantified the explosive activity from different viewpoints, in terms of intensity (i.e., jet elevation), dominant eruptive style (from ash emissions to coarse material jets) for each active vent, and average hourly explosive frequency per day (HEF), given as a total number, as well as for the NS and SCS separately. The intensity and frequency classification of the explosions adopted here is the same used for monitoring purposes at INGV-OE (Supplementary Table 1). The SPT was destroyed by the bombs of the 3 July paroxysm after recording only a few frames (Supplementary Movie 2); hence, the following activity including the 28 August paroxysm (Supplementary Movie 4) was evaluated continuously by the surviving SQT and SQV cameras at 400 m a.s.l. elevation. Single frames of the SPT and SQT cameras were extracted and after setting spatial scales (1.56 and 1.28 m per pixel, respectively), the vertical progression of the incandescent jet front in the first seconds after the explosions were measured frame by frame using ImageJ software (Supplementary Figs. 1–2).

### High-frequency thermal imaging

An evaluation of the explosive activity state preceding, in-between, and following the two paroxysmal events was performed in high frequency and resolution using high-speed thermal infrared videos of the explosive activity (Supplementary Movies 1, 3, 5). These were acquired from Pizzo (918 m a.s.l.) using a portable FLIR SC655 (640 × 480-pixel resolution at 50 Hz) on 9 May, 28 July, and 7 September 2019. One hour-long continuous sequences were analyzed to distinguish the active vents, classify the eruptive style (mainly puffing and magma jets) and evaluate the frequency and height of the explosions reached by bomb-sized material^61^.

### Field surveys

Field surveys were carried out on 5–7, 11–15, 26–28 July, and 1–7 September 2019, aimed at mapping tephra deposits and collecting representative samples to (i) map the isomasses of 3 July deposits, (ii), map the dispersal and size of juvenile and non-juvenile clasts, (iii) characterize texture and petrochemical composition of erupted products. Prompt field campaigns enabled us to map the 3 July tephra deposits after minimal reworking by wind and collecting more than 40 samples (25 representatives of the tephra fallout). In nine sites, 3–4 samples were taken within 1 × 1 m^2^ areas to evaluate the reliability of the deposit (i.e., if unaltered) and reduce the uncertainty in mass estimation. Additional samples were collected for further textural and petrochemical analysis on unmeasured surfaces, large leaves, or flat rocks.

Shortly after the 28 August paroxysm, prompt cleaning of the Stromboli urban areas together with heavy rains prevented a careful study of the primary fall deposit and estimation of mass loading. We collected samples for petrographic and compositional investigation from the coastline up to the summit and retrieved only a few, not weathered samples at low elevation for grain-size and textural analyses. However, we evaluated the fallout dispersal by the clasts found on the ground; over 17 sites between 220 m and 509 m a.s.l., we picked the largest clasts in areas of 50 m^2^, weighing the ten largest ones and measuring the average of the three orthogonal axes (in mm), and drew the isopleth map (Fig. 4 and Supplementary Table 3).

### Laboratory analyses

Tephra samples were processed and analyzed at INGV-OE Laboratory of Sedimentology (LS), INGV-PI Laboratory of Petrology and Volcanology (LPV), and INGV-RM1 High-Pressure High-Temperature Laboratory of Experimental Volcanology and Geophysics (HP-HT). Samples were dried in the oven at 60 °C and eventually weighed and/or sieved before being prepared for sedimentological, textural, and petrochemical analyses.

### Grain-size measurements and componentry analysis

The particle-size distribution of 19 tephra fallout samples (16 from 3 July; 3 from 28 August deposits, respectively) was determined by manual sieving at LS at 1 Φ intervals (Φ= −log_2_*d*, where *d* is the particle diameter in mm) from –5 to 6 Φ (64 mm to <0.032 mm). Componentry analysis was performed on P2 and P16 samples, by counting, under a stereomicroscope, about 200 particles, for the size intervals <−1 Φ, while, for 1 > Φ > −1 size interval, a 10 g aliquot of each size interval was washed in distilled water in an ultrasound bath, in order to remove any adhering fine ash. All clasts were observed for their macroscopic features (i.e., color, external shape, surface texture) to distinguish among juvenile glasses, crystals, and non-juvenile fragments. P16 was located along the main dispersal axis, P2 slightly offset to N.

### Lapilli bulk density and solid fraction density measurements

Thirty particles in the range 8–16 mm (−4 < Φ < −3, P02-1, P11-8, P13-2, for 3 July, P44, P62 for 28 August) and fifteen particles in the range 16–32 mm (-5 < Φ < -4, P33-01 for 3 July, P52-1, for 28 August) were selected to carry out density measurements at HP-HT. Samples were selected according to the dispersal axis: P02-1, P33-1, P44, and P52 were along the axis, whereas P11-8, P13-2, and P62 were lateral. The clast bulk or ‘envelope’ density ${\rho }_{{Pe}}$ (i.e., that referred to the volume of particles including both connected and closed vesicles) was determined following the method of Houghton and Wilson^62^, by weighing each particle in air and water. Clasts were wrapped with laboratory parafilm to prevent soaking and weighted using a high precision balance (Sartorius, 10^−4^ g) kit for density determinations. Clast envelope densities were converted to vesicularity values by $\chi =100\times \frac{{\rho }_{{{\rm{DRE}}}}{-\rho }_{{Pe}}}{{\rho }_{{{\rm{DRE}}}}}$, where ${\rho }_{{{\rm{DRE}}}}$ is the density of the solid fraction (dense rock equivalent, DRE) of the clasts measured with a Helium Pycnometer (Micromeritics AccuPyc II 1340, precision 10^−4^ cm^3^) on ca. 7–9 g of powdered clasts.

### Ash textural features

External morphology and groundmass texture were analyzed on 40–50 ash clasts randomly selected from the juvenile fraction of the samples P02-1 and P16-1 (3 July) and 19c (28 August) in the 1Φ (0.5–1 mm) size interval at LPV. The choice of this size class is a good compromise between representativeness and the capacity to observe textural variability in groundmasses since phenocrysts are most abundant in the larger size intervals. Moreover, in this size range, the vesicle and microlite content, size, and shape of the groundmass can be considered unaffected by post-fragmentation modifications considering the fast quenching in the air of the small particles. External morphology analysis was performed by imaging the particles arranged on a round glass (1 inch in diameter) covered by a carbon-coated tape, using the Zeiss EVO MA 10 Scanning Electron Microscope (SEM) at LPV and operating in Secondary mode. The round glass was then glued with epoxy resin and polished until exposing the inner portion of the clasts to investigate the textural and compositional features of the groundmass. Backscattered-Electron (BSE) images of single, unmingled, LP fragments were acquired at ×250 magnification and processed by means of Adobe Photoshop® software, for extracting vesicles that were then measured on binary images, using the ImageJ software. Type1 and Type2 vesicle size distributions were obtained by combining the high-resolution (3072 × 2304 pixels) images of 9 and 6 clasts from 3 July and 28 August samples, respectively. When the Type1 and Type2 populations coexist within single fragments, the smallest round vesicles (Type1) occupy the regions between large and elongated vesicles (Type2), these last resulting either from the coalescence of more than 2 bubbles in a preferential direction and by shearing during magma ascent. 3D vesicle size distribution was computed only on Type1 vesicles to avoid stereological problems during the conversion of the shape from 2D to 3D^28^, and also because they formed after the Type2 ones and thus represent a late stage of nucleation event in the shallow portion of the conduit.

### Petrochemical analyses

Ash, lapilli, and fragments of bombs were embedded in epoxy resin and polished for textural, mineralogical, and chemical investigations. Texture and chemical composition of minerals and groundmasses were analyzed at LPV using the Zeiss EVO MA 10 SEM equipped with an Oxford ISIS micro-analytical EDS system. The chemical analyses were performed at 15 kV, using a window of 10 × 10 µm to 20 × 20 µm for the glasses. The glass composition of the samples emitted on 3 July was also analyzed by EMPA at HP-HT, using a defocused beam, with a spot size of 5 μm (15 kV voltage, 5 nA beam current). The analytical errors, calculated using international standards, are between 2 and 5% for EMPA analyses, and generally lower than 5% for oxides with concentrations >5 wt%, lower than 10% for oxides between 1 and 5 wt% for EDS analyses.

### Eruption parameters

By analyzing video and images provided by video surveillance cameras, scientific personnel, tourists, and social networks^21^, we estimated the height and progression of the eruption plume of the 3 July paroxysm. 37 digital images and 18 digital videos, taken at distances between 0.5 and 45 km from the vents, were first collected and then georeferenced and spatially scaled by using visual cues. An absolute timeline of the videos and images was obtained by matching plume features in the videos and images that had a timestamp with those without it. Manual tracking using imageJ software of the plume front and of other noticeable features (including large eddies and plume irregularities) was then used to measure the time evolution of the plume. The tephra fallout mass of the 3 July paroxysm, was estimated based on the straight line exponential fit of Pyle^63^ by drawing 5 isomass lines (30, 4, 2.7, 1.7, and 0.2 kg m^−2^), and the Mass Eruption Rate (MER) by dividing the obtained mass by the estimated eruption time (20 s).

### Numerical modeling and inversion of the eruptive source parameters

A large set of simulations was performed by using the Tephra Transport Dispersal Model HYSPLIT^64^ initialized with the results of the eruptive column model PLUME-MoM^65,66^. The numerical wind data used to run the simulations was produced through the Weather Research and Forecasting Model^67^ to ensure the high spatial resolution necessary to properly model local tephra deposition on a small island like Stromboli. An Evolutionary Algorithm-based optimization was operated through the toolkit DAKOTA^68^. The simulated ground deposit mass was retrieved by finding the value that minimizes the difference between the simulated and the observed tephra fallout deposit. A full description of the procedures can be found in the Supplementary Methods.

## Supplementary information

Supplementary Information

Description of Additional Supplementary Files

Supplementary Movie 1

Supplementary Movie 2

Supplementary Movie 3

Supplementary Movie 4

Supplementary Movie 5

## Data Availability

The data that support the findings of this study are provided in the Supplementary Information file and available from the corresponding author upon reasonable request.

## Code availability

The numerical code for the plume and the scripts to couple it with Hysplit are available at https://github.com/demichie/PLUME-MoM-TSM. The digital object identifier (DOI) for the version of the code used in this paper is 10.5281/zenodo.3904379 (de’ Michieli Vitturi and Pardini, 2020).
