# Supplementary material for: Uncovering the eruptive patterns of the 2019 double paroxysm eruption crisis of Stromboli volcano
Source: Nat Commun. 2021 Jul 9;12:4213. doi: 10.1038/s41467-021-24420-1 (PMC8270928; doi:10.1038/s41467-021-24420-1)
Supplement: Supplementary file 1 — Supplementary Information [file 41467_2021_24420_MOESM1_ESM.pdf]

Supplementary Information for

Uncovering the eruptive patterns of the 2019 double paroxysm eruption crisis of Stromboli volcano

D. Andronico*, E. Del Bello*, C. D'Oriano, P. Landi, F. Pardini, P. Scarlato, M. de' Michieli Vitturi, J. Taddeucci, A. Cristaldi, F. Ciancitto, F. Pennacchia, T. Ricci, F. Valentini

*Corresponding authors. Email: daniele.andronico@ingv.it (D.A); elisabetta.delbello@ingv.it (E.D.B.)

This PDF file includes:

Supplementary Methods

Inversion of the eruptive source parameters

Supplementary Results

Vesicularity data on ash particles

Supplementary Figures 1 to 14

Supplementary Tables 1 to 4

References cited in this Supplementary Information file

Other Supplementary Material for this manuscript includes the following:

Supplementary Movies 1 to 5

Supplementary Methods

Inversion of the eruptive source parameters

A large set of simulations was performed by using the Tephra Transport Dispersal Model HYSPLIT[1]initialized with the results of the eruptive column model PLUME-MoM[2]. The numerical wind-data used to run the simulations was produced through the Weather Research and Forecasting Model[3] to ensure the high spatial resolution necessary to properly model local tephra deposition on a small island like Stromboli. An Evolutionary Algorithm-based optimization was operated through the toolkit DAKOTA[4].

Numerical models: PLUME-MoM and HYSPLIT

The release, dispersion and deposition of volcanic tephra for the paroxysm of 3 July 2019 at Stromboli was simulated through the coupled models PLUME-MoM[2] and HYSPLIT[1]. The former is an integral eruptive column model, while the latter is a transient 3D Volcanic Ash Tephra Transport and Dispersal model developed at NOAA Air Resources Laboratory. The two models are coupled in the way that the results of PLUME-MoM in terms of mass fluxes of solid particles lost from the column are used to initialize HYSPLIT. The coupling between the two codes is automated and based on Python routines, which automatically produce the HYSPLIT input files from the outputs of PLUME-MoM. A detailed description of this procedure can be found in[5,6].

For this work, we used the latest release of PLUME-MoM, named PLUME-MoM-TSM[7,8] , which includes new features such as particle aggregation and water phase changes. Moreover, PLUME-MoM-TSM has the possibility to simulate the umbrella cloud region by solving a set of shallow-water equations describing the initial lateral spread of the mixture above the neutral buoyancy height as a gravity current, also subject to drag exerted by the wind. The simulations done here do not account for aggregation and phase changes of water, while the umbrella cloud is simulated and used to initialize HYSPLIT. With respect to the procedure described in[5,6], here the coupling between PLUME-MoM-TSM and HYSPLIT was slightly modified to account for the umbrella cloud. In particular, below the neutral buoyancy level, tephra particles lost from the column enter the atmosphere at a number of heights located between the vent and the neutral buoyancy level. At each height, the injection sources are points positioned at the edge of the column. On the contrary, particles reaching the neutral buoyancy height enter the atmosphere from an area source, delimited by the circumference which at best fits the upwind part of the umbrella cloud.

Inversion settings

The inversion was performed by using the toolkit DAKOTA[4], an open-source software developed at Sandia National Laboratories, which allows the execution of analysis like Uncertainty Quantification, Sensitivity Analysis, inversion, and parameter estimation. Here, inversion was performed through a global optimization procedure by using an Evolutionary Algorithm (EA[9]) to find the best set of eruptive source parameters (ESPs) that minimize the difference between the simulated and observed tephra deposit. EAs are optimization methods based on Darwin's theory of survival of the fittest. They start the computation with a randomly selected population of design points in the parameter space (first generation). Thus, the best design points (i.e. those having low objective function values) survive and generate offspring (new generations) through selection, recombination, and mutation processes. EAs are widely used in engineering to locate the global optimum of complex optimization problems. The main drawback is that they need a great number of fitness function evaluations to converge.

The ESPs that we varied in the optimization procedure are: initial Total Grain-Size Distribution (TGSD), particle shape factor and column height (here we refer to the neutral buoyancy height). We discretized the mass distribution of the TGSD in 12 classes in phi scale from -5 to 6 phi (this range was set according to the phi classes observed in the deposit). For each size class, we searched for the

best fitting mass fraction in the range [0.001-1], while shape factors were optimized in ranges [0.1-0.5] for particles with size Φ<3 and [0.5-1.0] for finer particles, and column height in the range [2000m - 4000m] (for a given column height, PLUME-MoM automatically searches for the mass flow rate producing that height).

The objective function minimized by the EA was defined as:

$$f_{obj} = \sum_{i=1}^{P}\sum_{j=1}^{N}\frac{(o_{ij} - s_{ij})^2}{max[o_{ij}, s_{ij}]^2}$$

where *P* is the total number of tephra sample points, *N* is the number of classes forming the GSD of the i-th sample, and $o_{ij}$and $s_{ij}$are the sampled and simulated ground loads of the j-th class of the i-th sample, respectively. The square residuals are normalized using the maximum between the sampled and the simulated load.

We considered a total number of 16 sample points (P01-P16; Supplementary Fig. 9), each formed by 12 size classes (from phi -5 to phi 6). The location of such points is shown in Fig. 4. Supplementary Table 4 contains the input parameters of PLUME-MoM-TSM and HYSPLIT used to run the simulations. The EA algorithm was performed with 10000 objective functions evaluations and each generation of design points was formed by 600 individuals.

Supplementary Results

Vesicularity data on ash particles

Vesicles in pyroclasts are the remnants of the gas bubbles in magma, hence, their size, shape and distribution are a tool to quantify intra-conduit processes during eruptions. In particular, the smallest sized bubbles represent the last event in the processes of magma degassing, involving the nucleation, growth and coalescence of gas bubbles under non-equilibrium conditions during the ascent. Bubble texture in volcanic products of different kinds of eruption style has been studied by textural analyses of natural samples[10–12], theoretical modeling[13] and laboratory experiments[14–16]. A tendency of the number of vesicles per unit volume (N_V) to increase with eruption intensity has been experimentally and numerically linked to the dependence of N_V on decompression rate, and on other properties such as diffusivity, viscosity and surface tension[13,17–21]. There are many of these approaches related to Stromboli products, including those erupted during the paroxysms occurring in the past century[15] and in 2003 and 2007[10]. In these studies, the X-ray micro-tomography analysis of samples allows a direct estimation of the bubble volume distribution, while here we use a 2D approach, considering only bubbles with aspect ratio > 0.8 (Type1), almost spherical (circularity > 0.7; Supplementary Fig. 8). Vesicle size distribution was obtained by using CSD correction software[22,23]; as input data we select the major and minor axes and area of the best fit ellipse of measured bubbles, and as Reference Area, the area of melt, after subtraction of area of Type1 and Type2 bubbles. Elongated vesicles (Type2) were also considered for the 2D characterization.
Hereafter, we will extend the results shown in the main text (Physical features of the particles).

3 July paroxysm.

Type1 vesicles (N_A = 280 mm^{-2}) represent 13 vol.% of the bulk vesicularity (Φ), with a size ranging between 0.2 and 152 µm, where the most abundant sizes are between 6 and 25 µm. Vesicle volume distribution is characterized by a polymodal curve with at least two different modes, which can be correlated with distinct pulses of nucleation[24–26]. On the log-log plot of Nv>L vs. L (Fig. 6e), the CVSD follows an exponential law, and vesicle number density ($N_{V\ bulk}$) is $4.1*10^3$ mm^{-3}. In previous experiments[27], the exponential distribution has been correlated with a process of multiple events of bubble nucleation and growth (coalescence). Type2 vesicles (37 vol.%; N_A = 521 mm^{-2}) range in size between 0.5 and 667 µm, with the most abundant sizes around 20 µm. In some clasts, the large and elongated bubbles can coexist with the small and rounded ones. In all studied clasts and for all the size intervals, some bubbles show evidence of coalescence characterized by the interaction of generally two rounded vesicles, where the flat films developed between bubbles of the same size result markedly thinned and interrupted without retraction. Bubbles larger than 80 µm are the result of coalescence, as also evidenced by textural observations based on BSE images. It seems that the coalescence occurred during the ascent, when large and faster bubbles captured the new formed smallest ones (Ostwald ripening[28]), either at the wall conduit or within the rising magma column.

28 August paroxysm.

Type1 vesicles (N_A = 342 mm^{-2}) represent 11 vol.% of the whole vesicularity (Φ) and span in a range of size between 0.5 and 122 µm, with the most abundant sizes between 4 and 10 µm. Vesicle volume distribution (VVD) is characterized by a curve with at least three modes. However, on the other diagram, data point to a process of continuous/accelerating nucleation. In particular, on the log-log plot of Nv>L vs L (Fig. 6e), the CVSD follows a mixed exponential (small bubbles < 5 µm) and power law (large bubbles > 5 µm) distributions: this last with exponent d ∼ 2, and bubble number density (N_V) of $5.5*10^3$ mm^{-3}, confirming the above described trend. Type2 vesicles (N_A = 685 mm^{-2}) range in size between 1 and 365 µm, with the most abundant size (20 vol. % of all vesicles) ~16 µm. Similarly to the products of 3 July, the LP magma contains in the same ash grain both large, elongated and small, rounded bubbles, even if in the second paroxysm the Type2 bubbles tend to be smaller and more abundant than in the first one.

Supplementary Figures

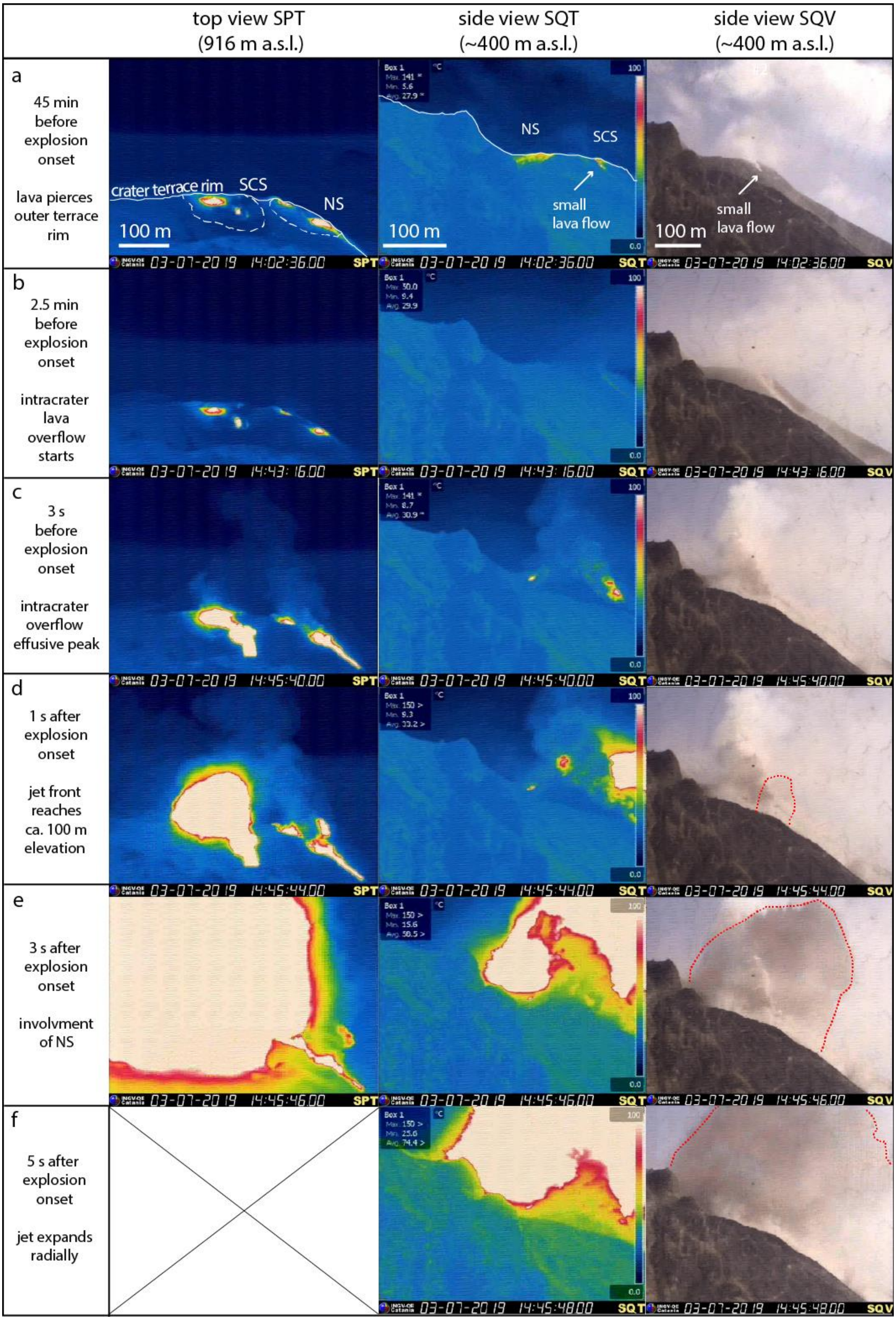

Supplementary Fig. 1. **Image sequence of the 3 July paroxysm from INGV surveillance video-cameras.** Each row shows simultaneous images from the thermal camera at Pizzo (SPT), thermal camera at 400 m (SQT), and visible camera at 400 m (SQV). a) Lava piercing of the wall terrace with lava flow output down the Sciara del Fuoco. b) Intracrater lava flow squeezing from the South Central Sector (SCS) and North Sector (NS) vents, and coeval stop on the lava at the Sciara del Fuoco. c) Maximum lava flow effusion rate from the SCS and NS vents. d) Onset of the paroxysmal sequence from the S vents. e) The following involvement of the NS vents 3s after explosion onset; the Pizzo thermal camera is transmitting the last few frames before being destroyed. f) The radial expansion of the jet is visible; the SPT camera has stopped to transmit new images. The red dotted lines in SQV d-f images mark the vertical progression of the incandescent jet front in the first 5 seconds after the explosion onset.

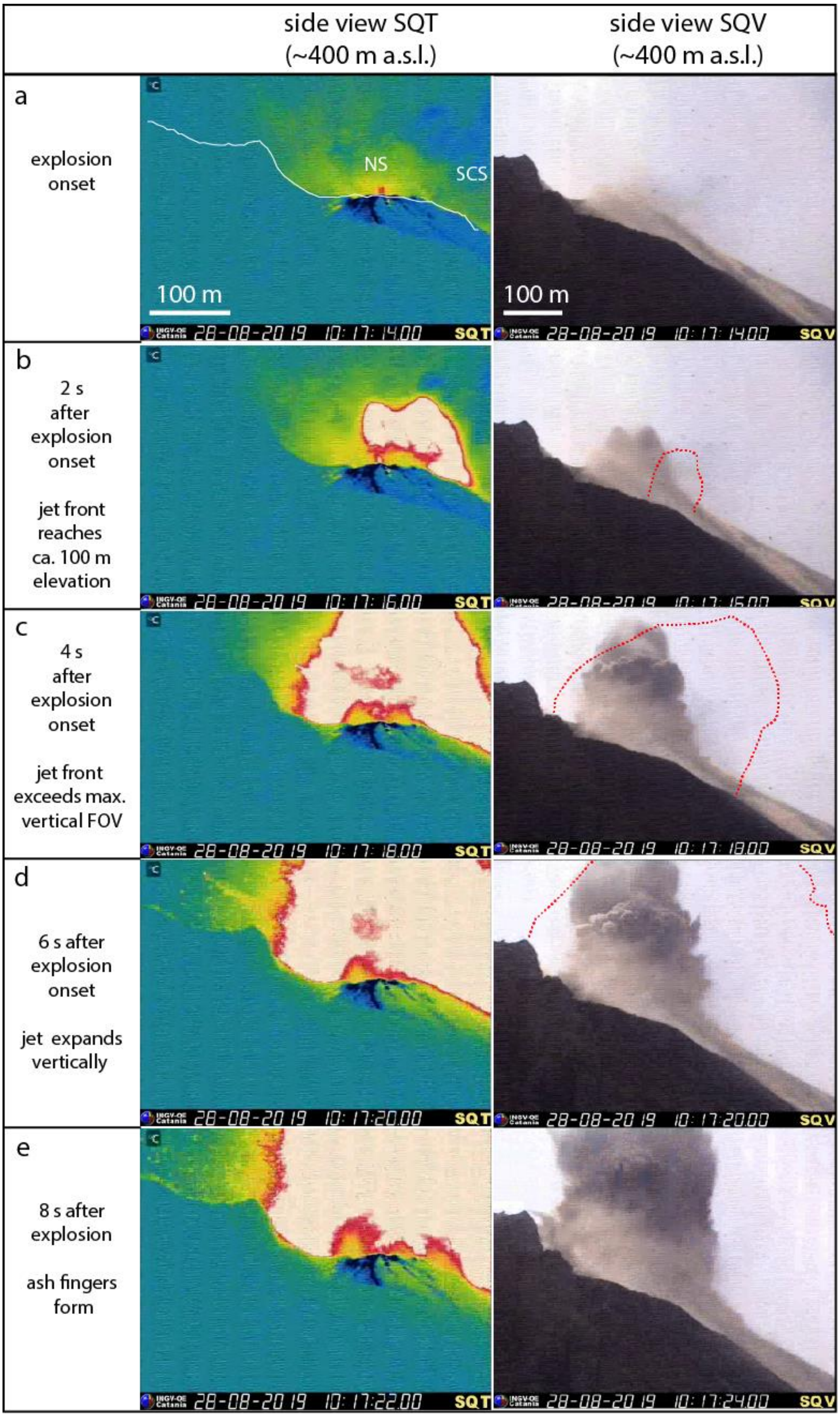

Supplementary Fig. 2. Image sequence of the 28 August paroxysm from INGV surveillance video-cameras. Each row shows simultaneous images from the thermal camera at 400 m (SQT) and visible camera at 400 m (SQV). South Central Sector (SCS) and North Sector (NS) vent areas are indicated. a) Onset of the paroxysmal sequence from the S vents. b-d) Vertical progression of the

incandescent jet front in the first 6 seconds after the explosion onset. b) The jet front reached 100 m above the vents 2 s after the explosion onset. c) The jet front exceeded the maximum field of view 4 s after the onset. d) The jet mainly expands vertically and exceeds the vertical FOV 6 s after the onset. e) Ash fingers formed from the jet front 8 s after the onset. For comparison, the contour of the jet front of the 3 July (red dotted line in b-d, as in Figure S1) is reported at the same time intervals after the explosion's onset.

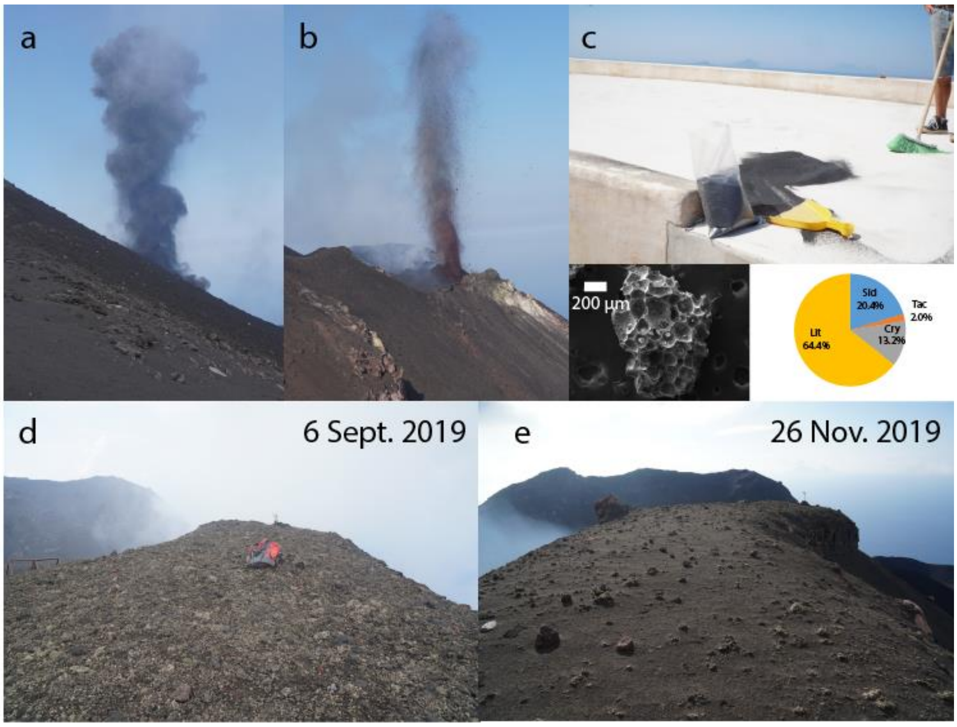

Supplementary Fig. 3. Eruptive activity post-3 July and post-28 August. a) Ash-rich explosion higher than 200 m from the SC sector. b) Strombolian scoria-rich explosion from the NS sector up to 150 m high. c) Ash removal from the roofs in Ginostra village. Inset: componentry of the abundant ash settled in Ginostra (Lit=Lithics, Sid=Sideromelane, Tac=Tachylite, Cry=Crystals), and SEM image of a sideromelane, HP particle. d-e) Comparison between the Pizzo area soon after the 28 August paroxysm (picture taken on 6 September, d) and after the intense ash explosions that lasted till mid-October 2019 (picture taken on 26 November, e), with an ash carpet ranging 10-15 cm in thickness. All photos by D. Andronico (a, b, c were taken on 26 July 2019).

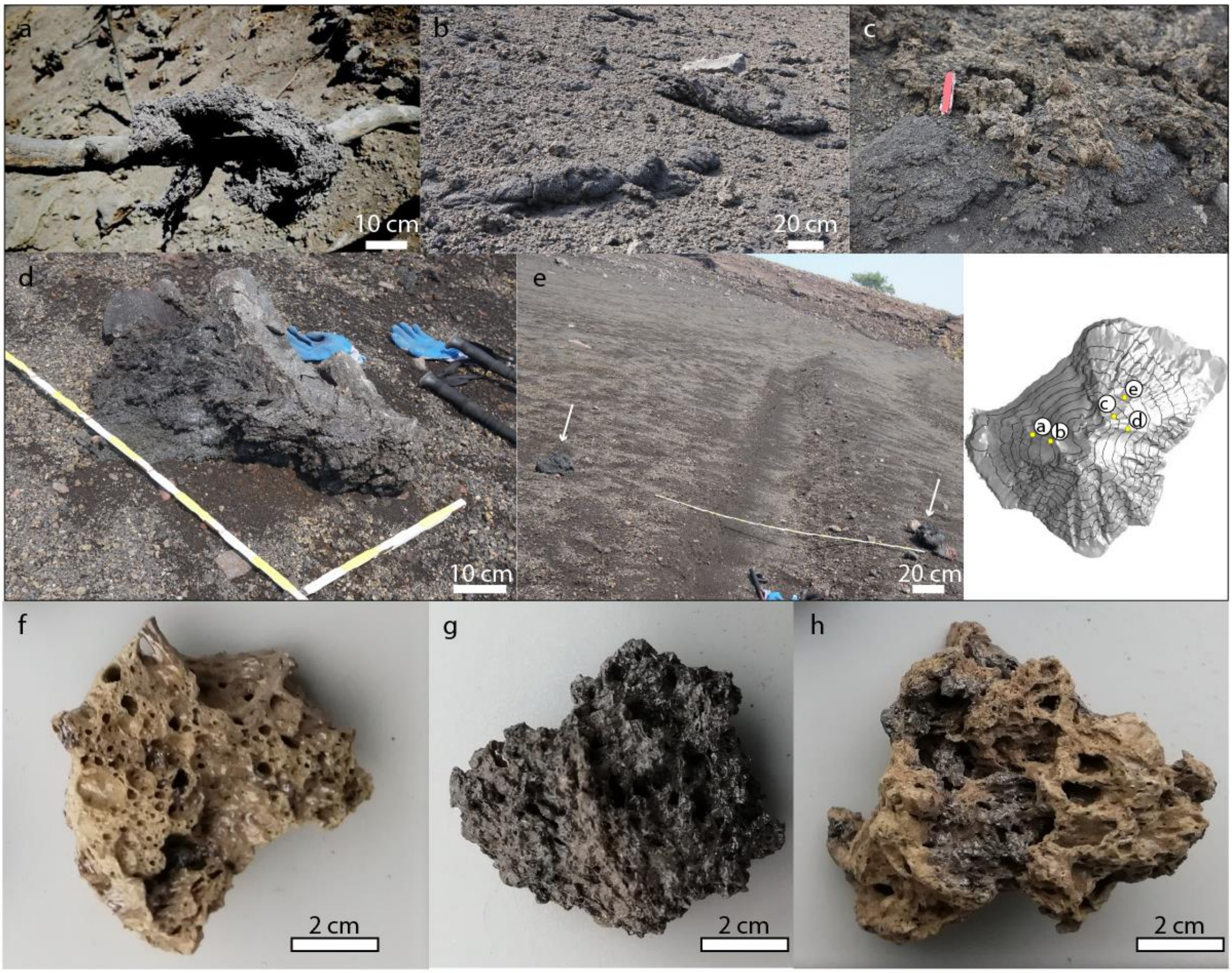

Supplementary Fig. 4. Ballistic fragments on the upper slopes of Stromboli. 3 July paroxysm: a) More than 60 cm-length, highly fluidal HP-scoria twisted around a tree branch, found at 500 m elevation in the W flank of Stromboli. b) Up to 2-m size, fusiform HP-scoria fallout in the Valle della Luna (400 m S of Pizzo). c) LP spatter bomb resting on HP metric-sized bomb. 28 August paroxysms: d) metric-sized HP bomb at about 600 m elevation at Liscione (E flank of Stromboli). e) Decimeter-sized non-juvenile and HP bombs fallen and partially rolled in the 400-m elevation tourist path (E flank). The map in the inset shows the location of the pictures. f-h) examples of an LP, an HP and a mingled HP-LP coarse lapilli. Photos a, b, d, e are by D. Andronico; photo c by P. Landi.

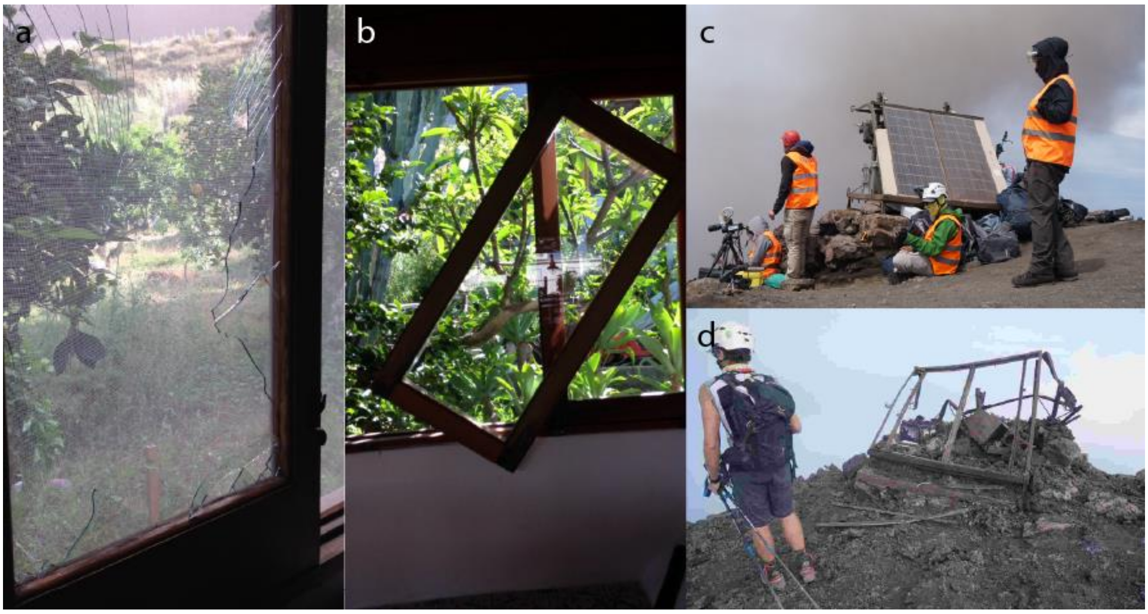

Supplementary Fig. 5. Some effects of the 3 July paroxysm. a) Shattered glass and b) blown off window frames at La Lampara restaurant – Stromboli village, more than 2 km from the vent, due to the shock wave (photo courtesy of Carlo Lanza, famed "pizzaiolo" and owner). c) The thermal camera monitoring station at Pizzo as it was on 9 May during an INGV field survey, and d) destroyed by ballistic fallout and shock wave after 3 July (photos c, d: D. Andronico).

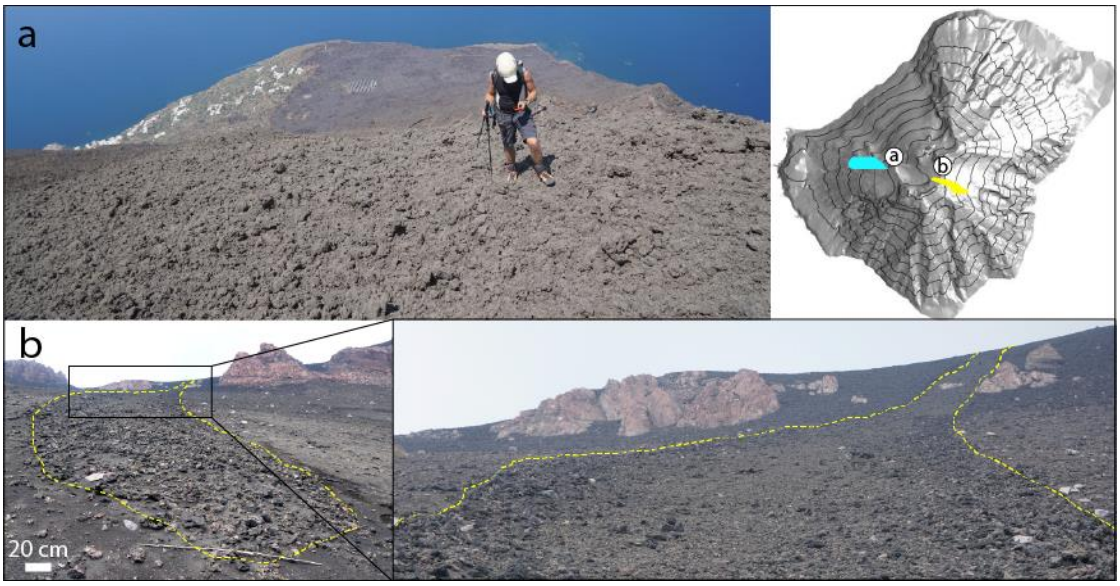

Supplementary Fig. 6. **Coarse tephra deposits emplaced during the 3 July 219 paroxysm**. a) Coarse-grained pumice deposit located on the upper W flank of Stromboli below the crater terrace. b) Gravity-induced flow on the opposite, E flank composed of up to 10-50 decimeter-size pomiceous bombs and non-juvenile fragments supported by abundant coarse-ash and sparse lapilli matrix. c) The front of the flow reaching 600 m above sea level. The map in the inset shows the approximate area of each deposit. Photos taken on 26 July 2019 (a) and 6 September 2019 (b, c) by D. Andronico.

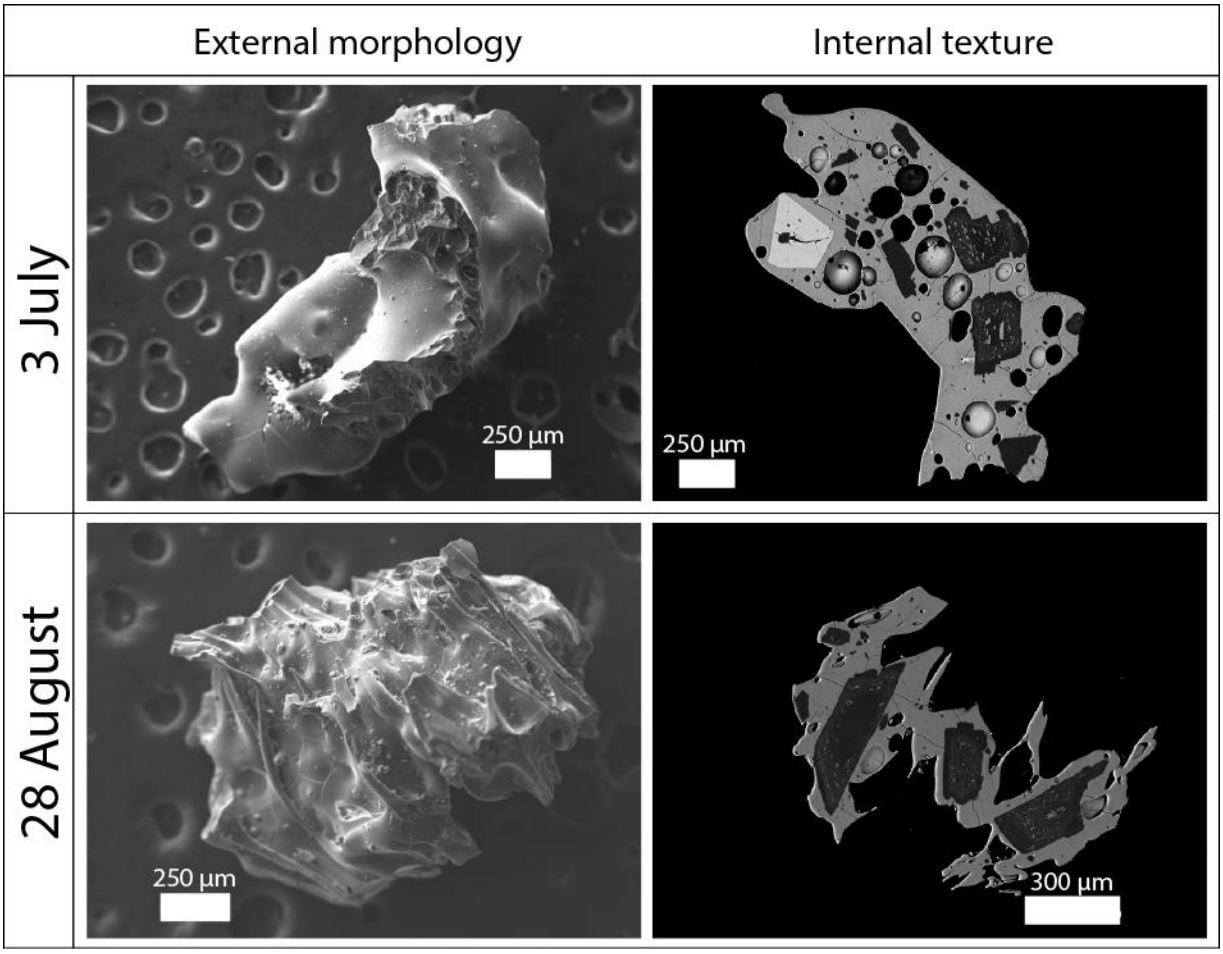

Supplementary Fig. 7. HP ash particles. SEM backscattered images of HP ash fragments from the two paroxysms. Most crystals are zoned plagioclase (dark crystals in the images on the right), associated with lower contents of clinopyroxene (not shown) and olivine (white crystal in the upper right image).

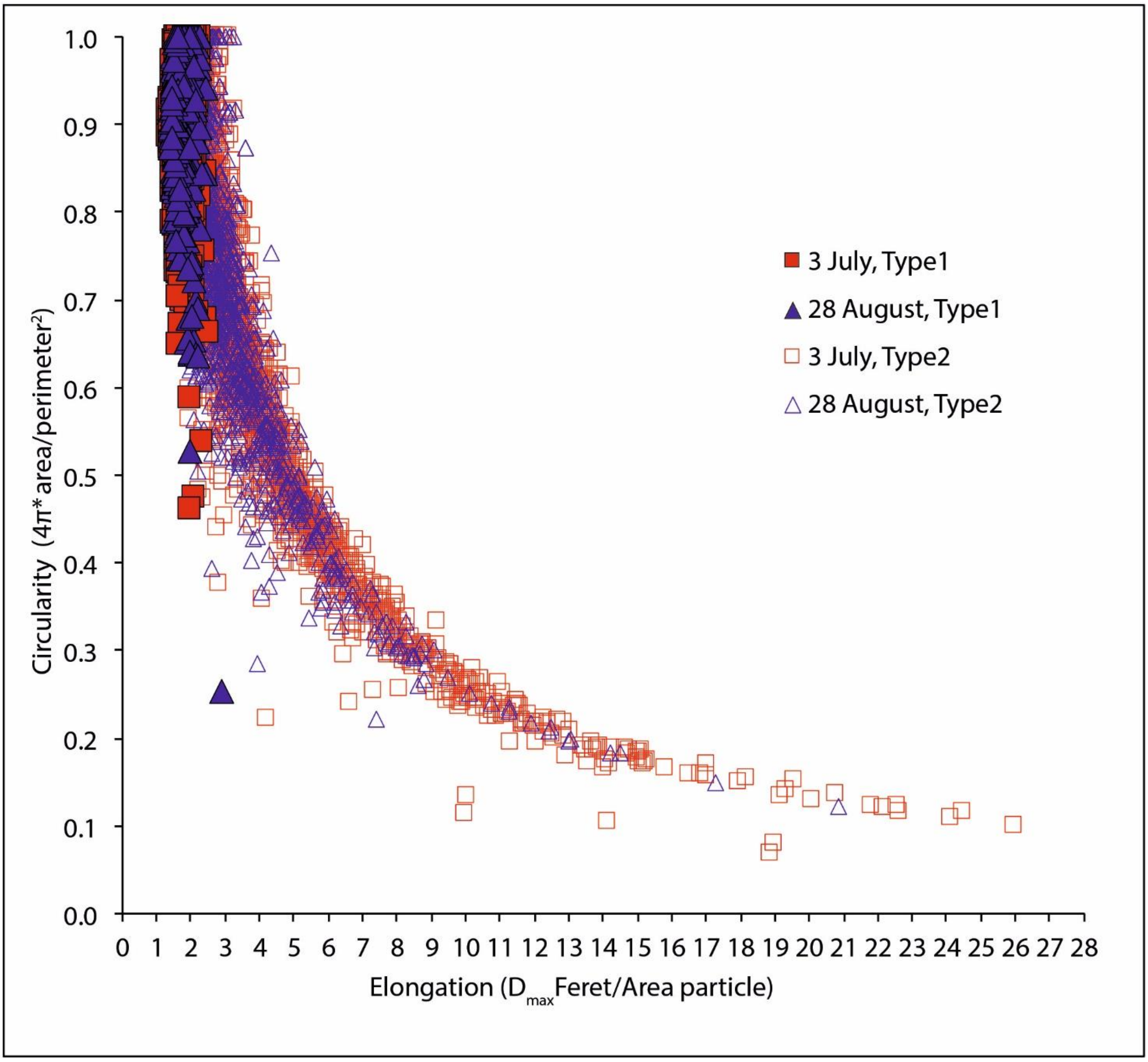

Supplementary Fig. 8. Circularity versus Elongation for the two types of vesicles detected in the studied samples. A Circularity value of 1 indicates a perfect circle. As the value approaches 0.0, it indicates an increasingly elongated shape. D_{max}Feret corresponds to the longest distance between any two points along the selection boundary. Type1 vesicles have Aspect Ratio (AR) > 0.8, and result less elongated (Elongation <2) and more rounded (Circularity > 0.7) than Type2 vesicles. Type1 vesicles are thought to be formed in the late stage of magma ascent, while Type2 vesicles result from elongation and shearing at the conduit walls, during magma ascent and/or to coalescence.

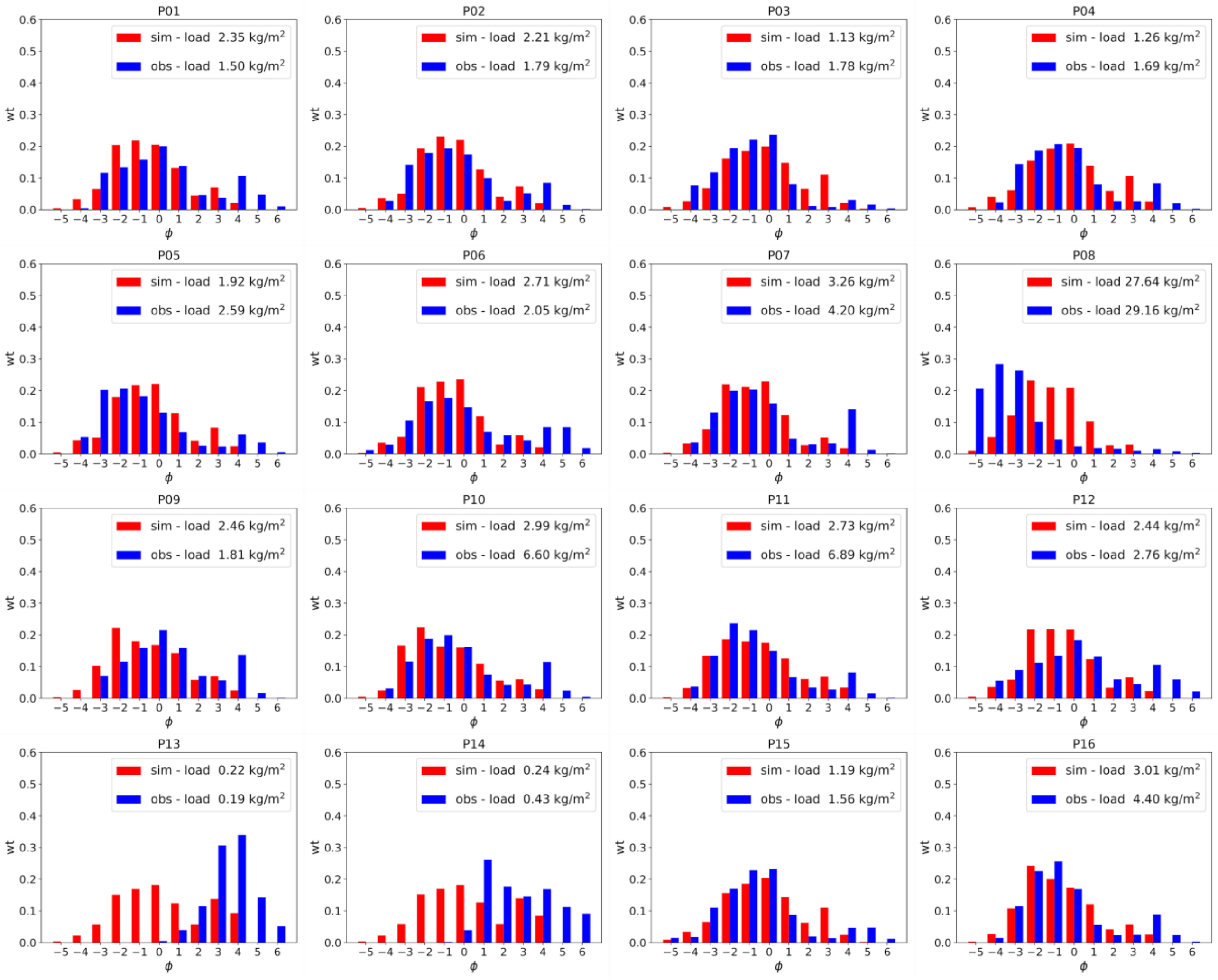

Supplementary Fig. 9. Simulated vs. observed grain-size distributions. Comparison between observed and simulated grain-size distributions for the 16 sample points.

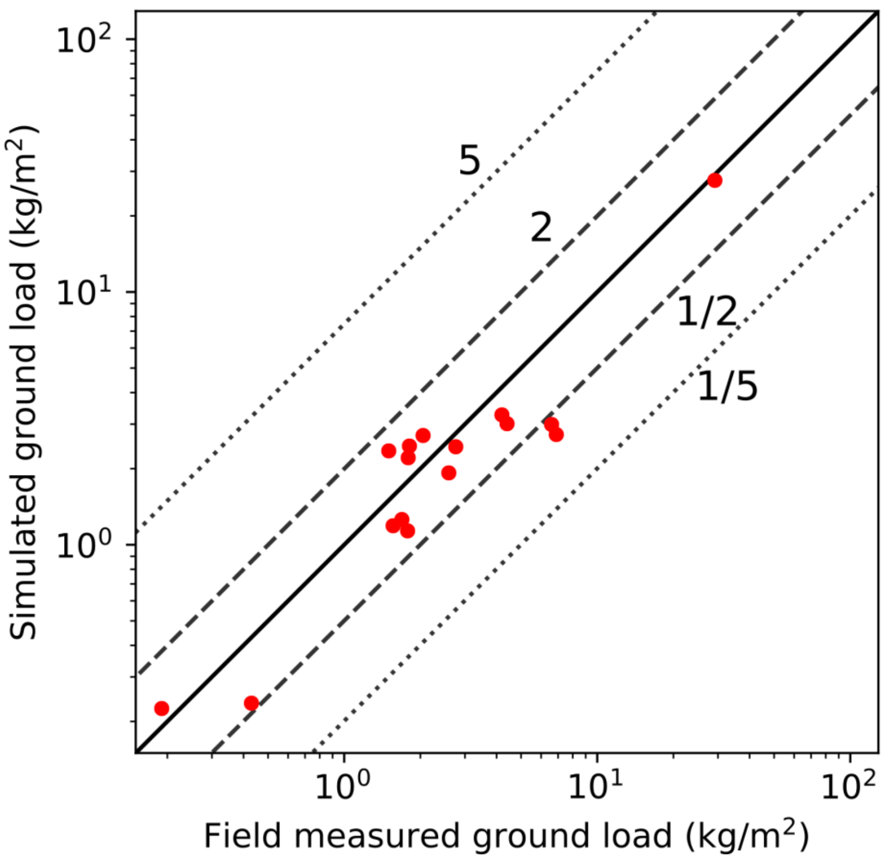

Supplementary Fig. 10. Simulated vs. observed ground loads. Comparison between the simulated and the observed ground loads at the 16 sample points. Dashed and point lines correspond to factors of 2 (1/2) and of 5 (1/5), respectively.

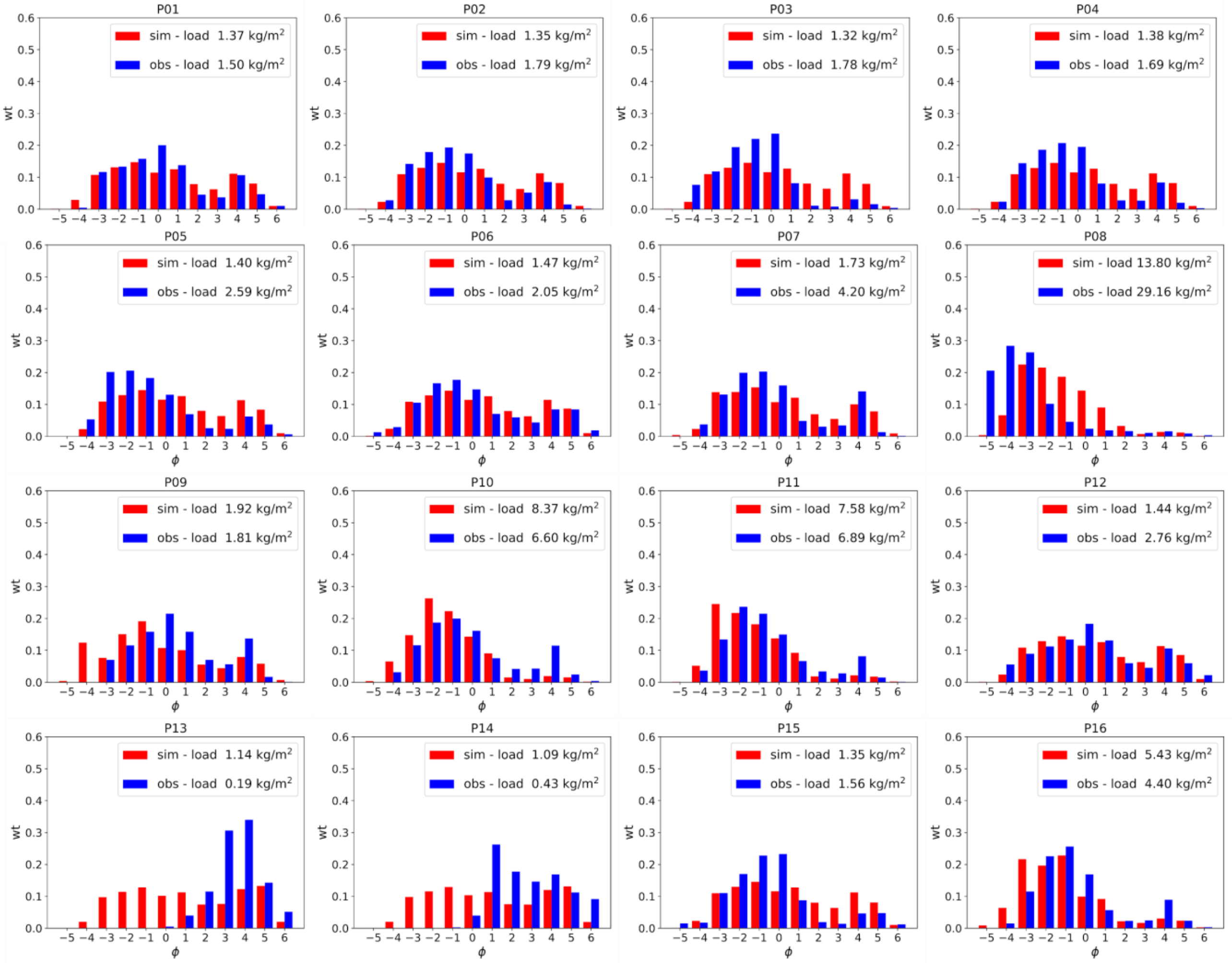

Supplementary Fig. 11 Observed and simulated grain-size distributions at the 16 sampling points. In the simulation, finer particles (Φ>3) were released from a source area located just above the vent instead of from the top of the column as usually done in standard model setting.

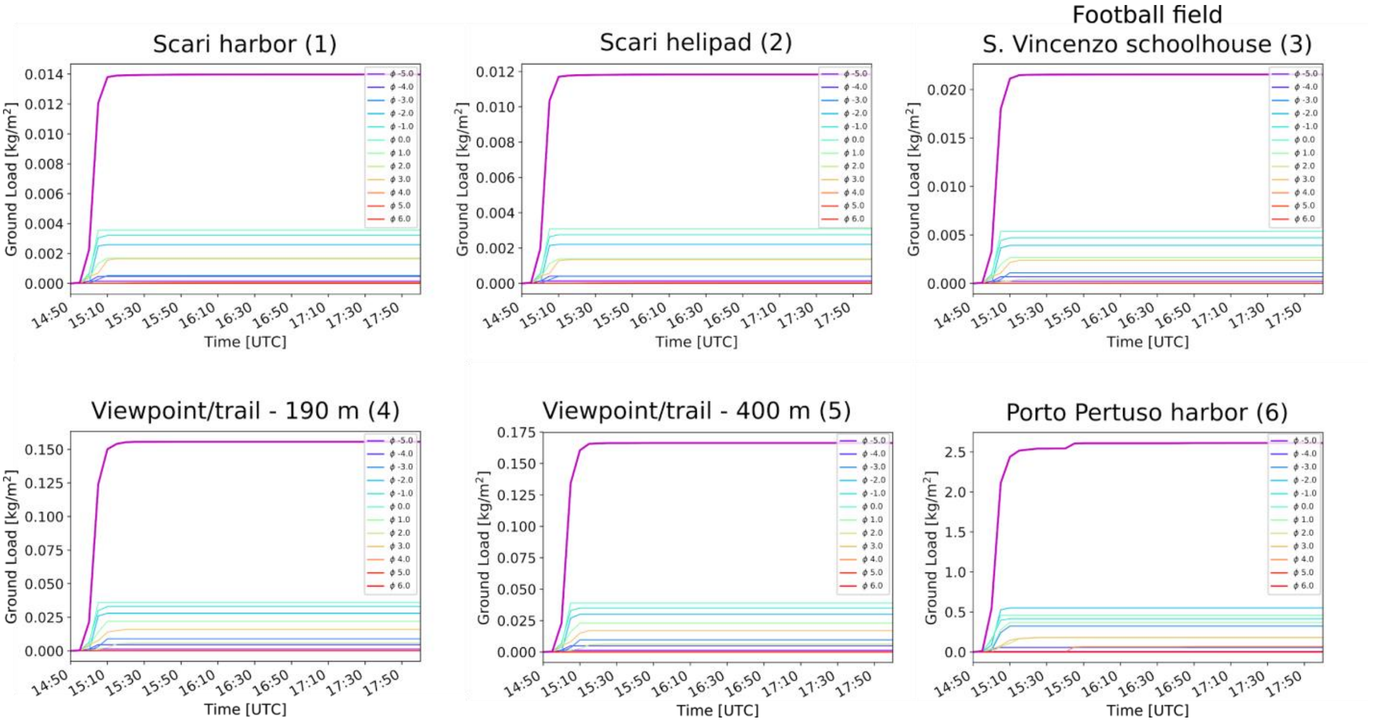

Supplementary Fig. 12. Simulated deposition time of tephra for 3 July. Deposition timings for the 6 high-risk locations resulting from the PLUME-MoM-TSM/HYSPLIT simulation for the 3 July case. Colored lines show the deposition timings of the particle classes forming the total grain-size distribution (TGSD, classes from Φ=-5 to Φ=6). The magenta line indicates the cumulative deposition timing considering all the classes.

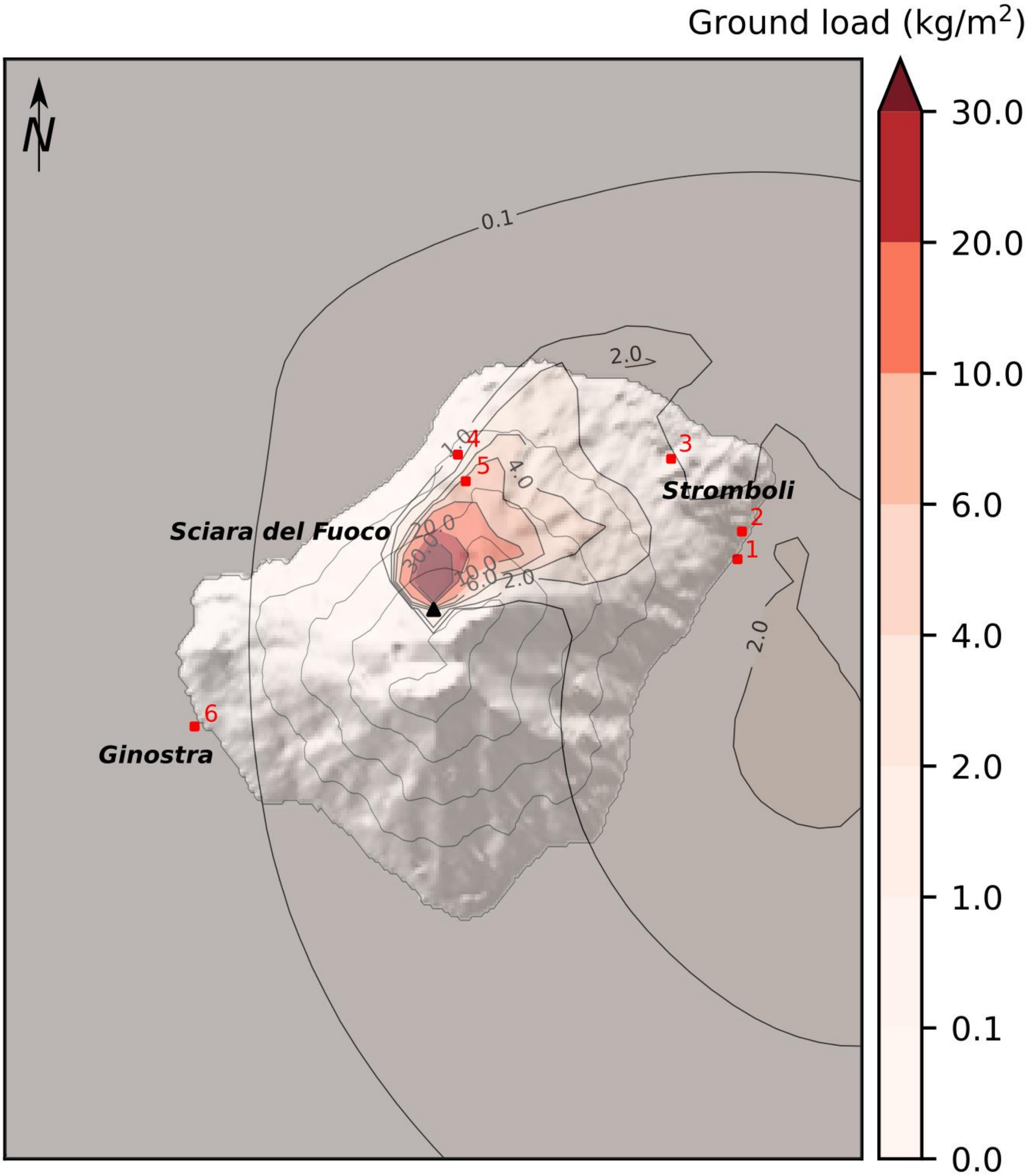

Supplementary Fig. 13. Worst-case simulated tephra deposit. Tephra deposit resulting from a PLUME-MoM-TSM/HYSPLIT simulation initialized with the input data used for the 3 July case, but with the wind-field rotated by 180° with respect to the original data. Differently from the 3 July case, the dispersion/deposition axis has a NE direction and the tephra fallout covers the village of Stromboli. A maximum load of about 70 kg m^{-2} is observed. Numbered red squares indicate the six high-risk locations of Stromboli, above Labronzo and Ginostra.

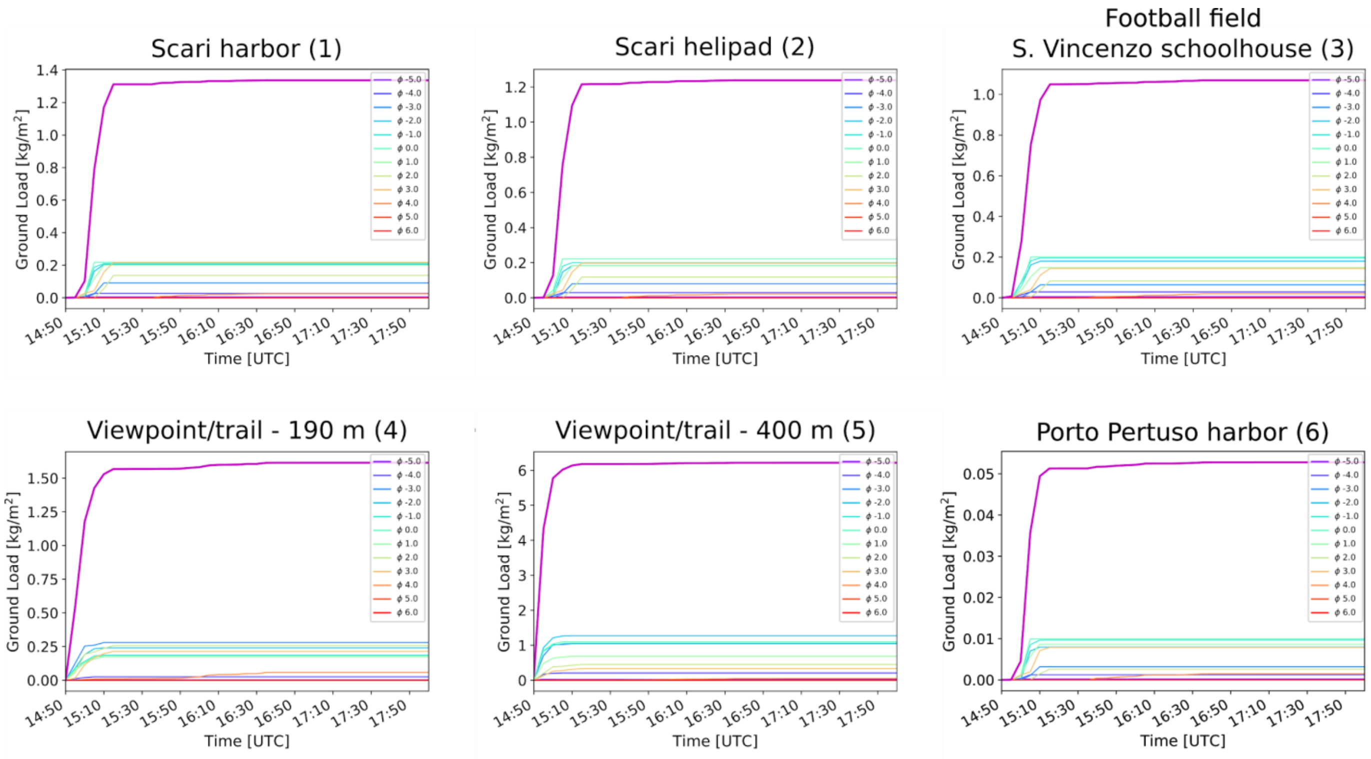

Supplementary Fig. 14. Worst-case simulated deposition time of tephra. Deposition timings for the 6 high-risk locations resulting from the PLUME-MoM-TSM/HYSPLIT simulation done with the 180° rotated wind.

Supplementary Tables

	**Explosion frequency** *Number of explosions per hour*	**Explosive intensity** *Bomb-sized clasts height above the terrace (m)*
**Low**	<5	<80
**Medium**	5-15	80-150
**High**	15-25	150-200
**Very high**	>25	>200

Supplementary Table 1. Classification of explosive intensity and frequency established for monitoring purposes at INGV-OE.

**Bomb size**			**Ground clast frequency**		
**n° clasts >40 cm**	19		***largest axis***	***area per clast*** m^2	***side of the square*** *m*
**maximum axis**	160 cm		>40 cm	4	2
**average of the largest axis of all clasts**	76 cm		>60 cm	5	2.3
**average of average of three axes of all clasts**	47 cm		>100 cm	16	4

Supplementary Table 2. Bomb size and ground-frequency at Helipad (total area 78.5 m^2 and located at 450 m distance from Pizzo) after the 3 July paroxysm.

**Number site**	**Elevation** m a.s.l.	**Latitude**	**Longitude**	**Cumulative weight** g *15 clasts*	**Average 3 axes** mm *10 clasts*
1	242	38°48'5.18"	15°13'37.45"	29	30
2	293	38°48'1.03"	15°13'36.49"	77	39
3	333	38°47'57.93"	15°13'34.57"	78	39
4	358	38°47'57.18"	15°13'32.45"	138	53
5	419	38°47'55.43"	15°13'26.92"	107	44
6	509	38°47'50.87"	15°13'20.97"	134	50
7	256	38°48'6.88"	15°13'31.78"	71	46
8	267	38°48'7.20"	15°13'29.32"	44	42
9	291	38°48'6.7"	15°13'27.0"	99	45
10	290	38°48'8.0"	15°13'22.3"	55	41
11	271	38°48'11.93"	15°13'19.88"	48	47
12	220	38°48'20.0"	15°13'14.3"	23	32
13	223	38°48'19.36"	15°13'10.09"	58	43
14	245	38°48'19.9"	15°13'6.1"	45	42
15	250	38°48'20.78"	15°13'2.72"	147	61
16	249	38°48'21.7"	15°12'57.9"	108	57
17	279	38°48'18.5"	15°12'50.0"	70	43

Supplementary Table 3. List of the 17 sites (and their coordinates) between 220 m and 509 m a.s.l. in the NE sector of Stromboli, where the biggest juvenile clasts from the 28 August tephra fallout were measured. For each site, the cumulative weight of the 15 largest clasts and the average of the 3 orthogonal axes of the 10 largest clasts are reported.

**PLUME-MoM-TSM/HYSPLIT input parameters**	
**Eruption start (UTC)**	2019/07/03 14:45
**Eruption end (UTC)**	2019/07/03 14:50
**Ash dispersion/deposition end (UTC)**	2019/07/03 18:00
**Vent lat (deg)**	38.793873
**Vent long (deg)**	15.211631
**Vent height (m)**	800
**Mixture temperature at the vent (K)**	1273
**Mixture velocity at the vent (m s^{-1})**	100
**Density part < -3 phi (kg m^{-3})**	700
**Density part > 3 phi (kg m^{-3})**	2700
**Specific heat (J Kg^{-1} K^{-1})**	1100
**Water mass fraction**	0.03
**Umbrella cloud drag coefficient**	1
**Umbrella cloud expansion duration (min)**	5
**Meteo data resolution (m)**	200
**Domain resolution (m)**	200
**HYSPLIT integration time step (min)**	1
**HYSPLIT output resolution (min)**	5
**HYSPLIT max number of puffs released**	1000000

Supplementary Table 4. Input parameters used for the numerical model PLUME-MoM-TSM/HYSPLIT.

References cited in this Supplementary Information file

1. Draxler, R. R. & Rolph, G. D. HYSPLIT (HYbrid Single-Particle Lagrangian Integrated Trajectory). *NOAA Air Resour. Lab. Coll. Park. MD* (2003).
2. De'Michieli Vitturi, M., Neri, A. & Barsotti, S. PLUME-MoM 1.0: A new integral model of volcanic plumes based on the method of moments. *Geosci. Model Dev.* **8**, 2447–2463 (2015).
3. Powers, J. G. *et al.* The weather research and forecasting model: Overview, system efforts, and future directions. *Bull. Am. Meteorol. Soc.* (2017) doi:10.1175/BAMS-D-15-00308.1.
4. Adams, B. M. *et al. DAKOTA, A Multilevel Parallel Object-Oriented Framework for Design Optimization, Parameter Estimation, Uncertainty Quantification, and Sensitivity Analysis Version 5.4 User's Manual SAND2010-2183*. *Sandia Technical Report SAND2010-2183* (2009).
5. Pardini, F. *et al.* Ensemble-based data assimilation of volcanic ash clouds from satellite observations: Application to the 24 december 2018 Mt. Etna explosive eruption. *Atmosphere (Basel).* (2020) doi:10.3390/atmos11040359.
6. Tadini, A. *et al.* Quantifying the Uncertainty of a Coupled Plume and Tephra Dispersal Model: PLUME-MOM/HYSPLIT Simulations Applied to Andean Volcanoes. *J. Geophys. Res. Solid Earth* (2020) doi:10.1029/2019JB018390.
7. de' Michieli Vitturi, M. & Pardini, F. demichie/PLUME-MoM-TSM: PLUME-MoM-TSM v1.0. *Zenodo* (2020) doi:10.5281/ZENODO.3904379.
8. de' Michieli Vitturi, M. & Pardini, F. PLUME-MoM-TSM 1.0.0: a volcanic column and umbrella cloud spreading model. *Geosci. Model Dev.* **14**, 1345–1377 (2021).
9. Bäck, T. *Evolutionary Algorithms in Theory and Practice: Evolution Strategies, Evolutionary Programming, Genetic Algorithms*. (Oxford University Press, Inc., 1996).
10. Polacci, M., Baker, D. R., Mancini, L., Favretto, S. & Hill, R. J. Vesiculation in magmas from Stromboli and implications for normal Strombolian activity and paroxysmal explosions in basaltic systems. *J. Geophys. Res. Solid Earth* **114**, 1–14 (2009).
11. Polacci, M., Corsaro, R. A. & Andronico, D. Coupled textural and compositional characterization of basaltic scoria: Insights into the transition from Strombolian to fire fountain activity at Mount Etna, Italy. *Geology* **34**, 201–204 (2006).
12. Shea, T. *et al.* Textural studies of vesicles in volcanic rocks: An integrated methodology. *J. Volcanol. Geotherm. Res.* **190**, 271–289 (2010).
13. Toramaru, A. BND (bubble number density) decompression rate meter for explosive volcanic eruptions. *J. Volcanol. Geotherm. Res.* **154**, 303–316 (2006).
14. Le Gall, N. & Pichavant, M. Homogeneous bubble nucleation in H_2O- and H_2O-CO_2-bearing basaltic melts: Results of high temperature decompression experiments. *J. Volcanol. Geotherm. Res.* (2016) doi:10.1016/j.jvolgeores.2016.10.004.
15. Le Gall, N. & Pichavant, M. Experimental simulation of bubble nucleation and magma ascent in basaltic systems: implications for Stromboli volcano. *Am. Mineral.* **101**, 1967–1985 (2016).
16. Blundy, J., Cashman, K. V., Rust, A. & Witham, F. A case for CO_2-rich arc magmas. *Earth Planet. Sci. Lett.* (2010) doi:10.1016/j.epsl.2009.12.013.
17. Mangan, M. & Sisson, T. Delayed, disequilibrium degassing in rhyolite magma: Decompression experiments and implications for explosive volcanism. *Earth Planet. Sci. Lett.* (2000) doi:10.1016/S0012-821X(00)00299-5.
18. Mourtada-Bonnefoi, C. C. & Laporte, D. Homogeneous bubble nucleation in rhyolitic magmas: An experimental study of the effect of H_2O and CO_2 . *J. Geophys. Res. Solid Earth* (2002) doi:10.1029/2001jb000290.
19. Mourtada-Bonnefoi, C. C. & Laporte, D. Kinetics of bubble nucleation in a rhyolitic melt: An experimental study of the effect of ascent rate. *Earth Planet. Sci. Lett.* (2004) doi:10.1016/S0012-821X(03)00684-8.

20. Namiki, A. & Manga, M. Influence of decompression rate on the expansion velocity and expansion style of bubbly fluids. *J. Geophys. Res. Solid Earth* (2006) doi:10.1029/2005JB004132.
21. Cluzel, N., Laporte, D., Provost, A. & Kannewischer, I. Kinetics of heterogeneous bubble nucleation in rhyolitic melts: Implications for the number density of bubbles in volcanic conduits and for pumice textures. *Contrib. to Mineral. Petrol.* (2008) doi:10.1007/s00410-8-0313-1.
22. Higgins, M. D. *Quantitative textural measurements in igneous and metamorphic petrology*. *Quantitative Textural Measurements in Igneous and Metamorphic Petrology* (2006). doi:10.1017/CBO9780511535574.
23. Higgins, M. D. CSD corrections Software 1.6. http://www.uqac.ca/mhiggins/csdcorrections.html (2018).
24. Klug, C., Cashman, K. & Bacon, C. Structure and physical characteristics of pumice from the climactic eruption of Mount Mazama (Crater Lake), Oregon. *Bull. Volcanol.* (2002) doi:10.1007/s00445-002-0230-5.
25. Polacci, M., Pioli, L. & Rosi, M. The Plinian phase of the Campanian Ignimbrite eruption (phlegrean fields, Italy): Evidence from density measurements and textural characterization of pumice. *Bull. Volcanol.* (2003) doi:10.1007/s00445-002-0268-4.
26. Lautze, N. C. & Houghton, B. F. Linking variable explosion style and magma textures during 2002 at Stromboli volcano, Italy. *Bull. Volcanol.* **69**, 445–460 (2007).
27. Blower, J. D., Keating, J. P., Mader, H. M. & Phillips, J. C. Inferring volcanic degassing processes from vesicle size distributions. *Geophys. Res. Lett.* **28**, 347–350 (2001).
28. Mangan, M. T. & Cashman, K. V. The structure of basaltic scoria and reticulite and inferences for vesiculation, foam formation, and fragmentation in lava fountains. *J. Volcanol. Geotherm. Res.* (1996) doi:10.1016/0377-0273(96)00018-2.
