# Supplementary material for: Uncovering the eruptive patterns of the 2019 double paroxysm eruption crisis of Stromboli volcano
Source: Nat Commun. 2021 Jul 9;12:4213. doi: 10.1038/s41467-021-24420-1 (PMC8270928; doi:10.1038/s41467-021-24420-1)
Supplement: Supplementary file 2 — Description of Additional Supplementary Files [file 41467_2021_24420_MOESM2_ESM.pdf]

Description of Additional Supplementary Files

File name: Supplementary Movie 1.
Description: This thermal video shows the explosive activity at the crater terrace of Stromboli on 9 May 2019 between 13:14 and 13:37 UTC. The explosions occur at 2 vents in the N sector (right side of the image), and in the S sector, where explosions occur within or at the side of a wide depression. In the C sector continuous degassing can be observed at two small pit-craters (in the foreground), while a hornito is emitting strong and audible particle-loaded jets. Video acquired at 50 Hz from Pizzo Sopra la Fossa by the HPHT volcanology group during a multidisciplinary data acquisition campaign.

File name: Supplementary Movie 2.
Description: The 3 July 2019 paroxysm as recorded by the INGV-OE surveillance cameras (thermal camera at Pizzo Sopra la Fossa at 918 m a.s.l.; visible and thermal cameras at 400 m of elevation).

File name: Supplementary Movie 3.
Description: This thermal video highlights the deep morphological changes that occurred three weeks after 3 July, due both to the 3 July paroxysm and the strong resumption of explosive activity at the crater terrace. Several vents were (often simultaneously) active in a wide area in the N sector, while a deep depression formed at the S sector. Video acquired at 50 Hz from Pizzo Sopra la Fossa on 28 July 2019 between 07:26 and 07:33 UTC by P. Scarlato.

File name: Supplementary Movie 4.
Description: The 28 August 2019 paroxysm as recorded by the INGV-OE surveillance cameras (visible and thermal cameras at 400 m of elevation).

File name: Supplementary Movie 5.
Description: This video was taken just ten days after the second paroxysm of 28 August, and shows that an intense Strombolian activity had built a small cone and a few hornitos at the N sector. In the S sector, the explosive activity involves a fissure-like area. Video acquired at 50 Hz and taken from Pizzo Sopra la Fossa on 7 September 2019 between 11:35 and 11:45 UTC by P. Scarlato and F. Pennacchia.
